# Raloxifene prevents stress granule dissolution, impairs translational control and promotes cell death during hypoxia in glioblastoma cells

**DOI:** 10.1038/s41419-020-03159-5

**Published:** 2020-11-17

**Authors:** Kathleen M. Attwood, Aaron Robichaud, Lauren P. Westhaver, Elizabeth L. Castle, David M. Brandman, Aruna D. Balgi, Michel Roberge, Patricia Colp, Sidney Croul, Inhwa Kim, Craig McCormick, Jennifer A. Corcoran, Adrienne Weeks

**Affiliations:** 1 Department of Surgery, Dalhousie University, Halifax, NS Canada; 2 Department of Medical Neuroscience, Dalhousie University, Halifax, NS Canada; 3 Division of Neurosurgery, Department of Surgery, Dalhousie University, Halifax, NS Canada; 4 Department of Pathology, Dalhousie University, Halifax, NS Canada; 5 Department of Microbiology & Immunology, Dalhousie University, Halifax, NS Canada; 6 Department of Biochemistry & Molecular Biology, University of British Columbia, Vancouver, BC Canada; 7 Department of Medicine, Dalhousie University, Halifax, NS Canada; 8 Department of Microbiology, Immunology and Infectious Disease, Cumming School of Medicine, University of Calgary, Calgary, AB Canada

**Keywords:** CNS cancer, Mechanisms of disease

## Abstract

Glioblastoma (GBM) is the most common primary malignant brain tumor, and it has a uniformly poor prognosis. Hypoxia is a feature of the GBM microenvironment, and previous work has shown that cancer cells residing in hypoxic regions resist treatment. Hypoxia can trigger the formation of stress granules (SGs), sites of mRNA triage that promote cell survival. A screen of 1120 FDA-approved drugs identified 129 candidates that delayed the dissolution of hypoxia-induced SGs following a return to normoxia. Amongst these candidates, the selective estrogen receptor modulator (SERM) raloxifene delayed SG dissolution in a dose-dependent manner. SG dissolution typically occurs by 15 min post-hypoxia, however pre-treatment of immortalized U251 and U3024 primary GBM cells with raloxifene prevented SG dissolution for up to 2 h. During this raloxifene-induced delay in SG dissolution, translational silencing was sustained, eIF2α remained phosphorylated and mTOR remained inactive. Despite its well-described role as a SERM, raloxifene-mediated delay in SG dissolution was unaffected by co-administration of β-estradiol, nor did β-estradiol alone have any effect on SGs. Importantly, the combination of raloxifene and hypoxia resulted in increased numbers of late apoptotic/necrotic cells. Raloxifene and hypoxia also demonstrated a block in late autophagy similar to the known autophagy inhibitor chloroquine (CQ). Genetic disruption of the SG-nucleating proteins G3BP1 and G3BP2 revealed that G3BP1 is required to sustain the raloxifene-mediated delay in SG dissolution. Together, these findings indicate that modulating the stress response can be used to exploit the hypoxic niche of GBM tumors, causing cell death by disrupting pro-survival stress responses and control of protein synthesis.

## Introduction

Glioblastoma multiforme (GBM) is the highest grade (IV) and most common primary brain tumor. GBM can occur de novo or from related lower-grade counterpart astrocytomas (Grade II/III); although all lower-grade astrocytomas will progress to GBM. Lower-grade astrocytomas are slow growing lesions characterized by the absence of both necrosis and vascular proliferation which are pathognomonic of GBM. The current treatment paradigm (surgery, radiation, and temozolomide) has a dismal 25% 2-year survival rate^1^. Treatment failures occur at the primary site of occurrence, thus relapse fundamentally results from treatment resistance.

Tight regulation of protein synthesis is required to meet, but not exceed, the anabolic demands of the cell. In response to environmental stress, protein synthesis is rapidly arrested to prevent production of aberrant proteins and promote cell survival and restoration of homeostasis. There is strong evidence that this type of translation control plays an important role in cancer progression^2–5^. Cancer cells place higher demands on the protein synthesis machinery, ramping up translation and subverting regulatory mechanisms. For example, GBM cells thrive in a stressful, low oxygen environment and survive radiation doses that would normally arrest protein synthesis and halt cell proliferation^6^. GBM is thought to recur because GBM cells can alter translational landscapes and survive hypoxic, radiated, and chemotherapeutic microenvironments^7,8^. Understanding mechanisms of translation control in GBM cells could enable the identification of new therapeutic targets.

Stress granules (SGs) are cytoplasmic, non-membrane-bound foci of mRNAs and RNA-binding proteins that form to spatially regulate mRNA stability, localization, and translation during the integrated stress response (ISR). SGs form in response to activation of at least one of the four stress-sensing sentinel kinases: protein kinase R (PKR), PKR-like endoplasmic reticulum kinase (PERK), general control nonderepressible 2 (GCN2), and heme-regulated eukaryotic translation initiation factor 2 alpha kinase (HRI). These kinases phosphorylate eukaryotic translation initiation factor 2 alpha (eIF2α), which increases binding affinity between eIF2α and the guanine nucleotide exchange factor eukaryotic translation initiation factor 2B (eIF2B), thereby blocking the recharging of the eIF2–GTP–tRNA^iMet^ ternary complex and preventing translation initiation^9,10^. Bulk translationally stalled mRNAs are bound by aggregation-prone proteins with intrinsically disordered domains (e.g T-cell-restricted intracellular antigen-1 (TIA-1), TIA-1-related protein (TIAR), and Ras GTPase-activating protein-binding protein 1/2 (G3BP1/2)) that drive SG aggregation^11^. However, this inhibition of bulk translation is incomplete, and SGs promote cell survival by re-prioritizing the translational apparatus to the generation of proteins that facilitate stress adaptation and cell survival. SGs also allow rapid resumption of protein synthesis following stress resolution, since mRNAs released from SGs are already bound to translation initiation machinery^5^.

SGs are highly dynamic structures that in physiologic conditions dissolve even in the persistence of stress^12^. A key step is the dephosphorylation of eIF2α, which recharges the eIF2–GTP–tRNA^Met^ ternary complex and allows re-entry in translation. Dephosphorylation can occur by the constitutively active reverter of eIF2α phosphorylation (CReP) or by the stress-induced phosphatase, growth arrest, and DNA damage-inducible protein (GADD34)^13^. However, timely dissolution of SGs requires functioning protein quality control (PQC). Severe stress increases the content of misfolded protein and defective ribosomal products (DRiPs). The aggregation of DRiPs and misfolded proteins into SGs results in SGs that are difficult to dissolve^14,15^. In instances where the stress overwhelms the PQC, SGs persist. Granulostasis would then affect the ability of cells to properly restore translation after stress, with potentially harmful effects and loss of cellular viability^16^.

Hypoxia is one of the canonical stressors that triggers SGs by activating PERK/PKR/GCN2^17–19^. GBM, like many solid tumors, features extensive hypoxic regions. GBM tumor cells must have mechanisms to thrive despite the normally cytostatic/cytotoxic effects of hypoxia. The tightly regulated formation and dissolution of SGs is a key mechanism that allows cells to endure environmental stress. Therefore, we hypothesized that hypoxia-induced SGs play a significant role in GBM pathobiology. Importantly for cancer research, SGs can be pharmacologically manipulated, making SGs potential targets for therapy^20–24^. Utilizing high-throughput image analysis and the Prestwick Chemical Library, we screened 1120 compounds for the ability to disrupt SG dynamics. We discovered that selective estrogen receptor modulators (SERMs) including raloxifene prevent disassembly of hypoxia-induced SGs and promote cell death.

## Materials and methods

### Cancer Genome Atlas interrogation

The Cancer Genome Atlas (TCGA) was interrogated with GlioVis^25^, www.gliovis.bioinfo.cnio.es in February 2019 for *PERK*, *GCN2*, *G3BP1*, *eIF2α*, and *GADD34* mRNA expression and survival in low-grade astrocytoma and GBM. Statistical analysis (Tukey’s honest significant difference and log rank *p*-values) was determined using the statistical package included in the software. Corrections for multiple comparisons were made in both the expression data and Kaplan–Meier curve data.

### Immunohistochemistry

A tissue microarray (TMA) consisting of 90 intact GBM cores from 45 patients and two normal cortex controls was obtained from Dr. Sidney Croul in the Department of Pathology at Dalhousie University. The TMA was serial sectioned and prepared for immunohistochemical labeling as previously described^26^. Tissue sections were labeled with primary antibodies: TIAR (BD Biosciences, 610352; diluted 1:60), G3BP2 (Sigma-Aldrich, HPA018304; diluted 1:1000), hematoxylin and eosin. Cytoplasmic staining was graded with the help of a pathologist. Cytoplasmic granular staining was graded from 0 to 4; 0 being no cytoplasmic granular staining, 1 being minority of cells with cytoplasmic granular staining, 2 being ~50% of the cells with cytoplasmic granular staining, 3 being the majority of cells demonstrating cytoplasmic granular staining and 4 being all the cells (vascular endothelium excepted) exhibiting cytoplasmic granular staining.

### Cell lines and cell culture

Immortalized human GBM U251 MG cells (a generous donation from J. Rutka; Sigma-Aldrich origin) and HEK293T cells (ATCC) were cultured in Dulbecco’s modified Eagle’s medium (DMEM) (high glucose, no sodium pyruvate) supplemented with 1% glutamine, penicillin–streptomycin, and 10% fetal bovine serum (Gibco). Primary U3024 MG (HGCC RRID:CVCL_IR67) cells were cultured on polyornithine and laminin-coated plates in 1:1 neurobasal and DMEM/F12 glutamax media supplemented with 1% penicillin–streptomycin, B-27, N-2 (Gibco), 10 ng/mL epidermal growth factor (EGF), and 10 ng/mL fibroblast growth factor (FGF) (Peprotech). All cells were cultured at 37 °C with 5% CO_2_ and negative for mycoplasma.

### Drug screen

A drug screen was conducted using the Prestwick Chemical Library (Prestwick Chemical, Illkirch, France); a collection of 1120 drugs and small molecules, 95% of which are approved for use in humans (FDA, EMA, and other agencies). U251 cells were seeded at a density of 75,000 cells/mL and treated with 30 μM of each drug for 1 h at 37 °C and subjected to 2 h of hypoxia (<1% O_2_) using a hypoxia incubator chamber (see below). Cells were fixed and stained for the SG marker TIAR 1 h post-release from hypoxia to identify drugs interfering with SG dissolution. A second drug library was applied to U251 cells for the same time course but in normoxia. Plates were read using a Cellomics Arrayscan V^TI^ automated fluorescence imager. Images were analyzed with Arrayscan software (Zeiss) and SGs counted by the ArrayScan V^TI^ HCS Reader algorithm^27^; data was exported to and analyzed with Microsoft Excel. Drugs were considered positive hits if the mean number of granules per cell was two standard deviations (SD) from the mean of control cells. All 1120 drugs were ranked according to their *z*-scores (SGs present at 1 h normalized to non-drug controls). Final drug selection was based on a combination of deviation from control mean SGs per cell and literature linking them to either GBM treatment or a role in hypoxic or oxidative stress. None of the top candidate drugs were linked to SGs prior to our selection.

### Hypoxia

U251 cells were seeded at a density of 100,000 cells/mL and treated with a final concentration of 40 μM raloxifene hydrochloride (Cayman Chemicals), 40 μM chloroquine (CQ) (Sigma-Aldrich) or dimethyl sulfoxide (DMSO) vehicle control for 1 h at 37 °C. Hypoxia (<1% O_2_) was induced using a hypoxia incubator chamber (STEMCELL Technologies). Immediately prior to placing cells in the hypoxia chamber, half of the media was removed from each well to limit the presence of dissolved oxygen. For control cells that did not undergo hypoxia, the same amount of media was removed but the cells were not placed in the hypoxia chamber and remained at 20% O_2_ (normoxia). To induce hypoxia, the chamber was flushed with high purity (99.9%) nitrogen gas at 2 psi for 10 min, sealed and incubated at 37 °C for 50 min. This was followed by a second 10 min nitrogen gas flush and a 1 h incubation at 37 °C (for a total of 2 h of hypoxia). U3024 MG primary cells were treated with raloxifene or DMSO vehicle control in serum-free DMEM (as Neurobasal media contains sodium pyruvate which prevents the formation of hypoxia-induced SGs; see Supplementary Fig. 4). U3024 cells received a total of 1 h hypoxia (10 min nitrogen gas flush plus 50 min incubation in the hypoxia chamber).

### Immunofluorescence

Following hypoxia or the equivalent normoxia incubation, cells were fixed at various times in 4% paraformaldehyde for 10 min. Following fixation, outer cellular membranes were stained with wheat germ agglutinin (WGA), alexa 647 conjugate (Thermo Fisher Scientific) diluted 1:400 in PBS for 10 min and permeabilized in 0.1% Triton X-100 for 10 min. Cells were blocked in 8% BSA in PBS for 1 h followed by a 1 h incubation with antibodies specific for TIAR (BD Biosciences, 610352; diluted 1:200), G3BP2 (Sigma-Aldrich, HPA018304; diluted 1:1500), or FMRP (Cell Signaling Technology, 7104; diluted 1:400) in 1% BSA. Subsequently, cells were incubated with Alexa Fluor 488-chicken anti-mouse and Alexa Fluor 555-donkey anti-rabbit (Thermo Fisher Scientific) secondary antibodies in 1% BSA for 1 h. Cell nuclei were counterstained with 1 μg/ml 4′,6-diamidino-2-phenylindole (DAPI, Sigma-Aldrich). Coverslips were mounted on frosted glass microscope slides using ProLong Gold antifade mounting media (Thermo Fisher Scientific) and allowed to cure overnight.

### Quantification of SGs

*Imaging*: Imaging was performed on an AxioImager Z2 (Zeiss) microscope at ×40 objective. Ten fields of view were obtained per timepoint ± hypoxia, imaging 100+ cells per condition. Exposure on all channels was unchanged through the imaging and intensity ranges of each image were identical. Images were exported as separate channel TIF files and quantified using CellProfiler.

*CellProfiler*: Image analysis was performed using CellProfiler (cellprofiler.org), an open source software for image analysis^28^. The pipeline used was designed as follows. Nuclei were detected as primary objects using automatic thresholding of the DAPI image. Cells were identified as secondary objects using the propagate function from the identified nuclei, determining the cell’s outer edge in the WGA image. Following a series of enhancement and masking steps, puncta were measured in the cytoplasm of cells using a global thresholding with robust background adjustments. Puncta number per cell, intensities and locations were measured and exported as.csv files.

*R analysis:* RStudio software was used to analyze CellProfiler output. Puncta counts per cell from two SG markers were determined and only puncta that had sufficient intensity measurements and >50% correlation from both markers were considered SGs. Error and significance of puncta counts within a given experiment was determined using a negative binomial model, commonly used for count data with unequal variance.

### Western blot analysis

Total cell lysates were harvested in Laemmli lysis buffer and protein concentration determined using the DC Protein Assay (Bio-Rad). Protein lysates were separated in 12% TGX stain-free gels which were then activated for 45 s after SDS-electrophoresis, transferred to PVDF membranes using the Trans-Blot Turbo transfer system and imaged with the ChemiDoc Touch imaging system (Bio-Rad). Primary antibodies were used as follows: mouse anti-puromycin (1:8000; EMD Millipore, MABE343), rabbit anti-eIF2α (1:1000; Cell Signaling Technology, 9722), rabbit anti-phospho-eIF2α (Ser51) (1:500; Cell Signaling Technology, 9721), rabbit anti-ribosomal protein S6 (rpS6) (1:4000; Cell Signaling Technology, 2317), rabbit anti-phospho-rpS6 (Ser235/236) (1:4000; Cell Signaling Technology, 2211), rabbit anti-GADD34 (1:750; Thermo Fisher Scientific, PA1-139), rabbit anti-LC3B (1:1000; Cell Signaling Technology, 2775), mouse anti-SQSTM1/p62 (D5L7G) (1:1000; Cell Signaling Technology, 88588), mouse anti-G3BP1(1:250, Santa Cruz Biotechnology, sc-81940), rabbit anti-G3BP2 (1:2500; Sigma-Aldrich, HPA018304). Detection was performed with peroxidase-coupled secondary antibodies (Cell Signaling Technology) with Clarity Western ECL substrate (Bio-Rad). All blots were normalized to total lane protein and band intensities were quantified using ImageLab software (Bio-Rad).

### Annexin/PI flow cytometric analysis

The Annexin V-Alexa Fluor 488/propidium iodide (PI) dead cell apoptosis kit (Thermo Fisher Scientific V13241) was used to detect early and late apoptosis and necrosis. Cells were treated with increasing doses of raloxifene (40–100 μM) and 2 h following hypoxic or the equivalent normoxic incubation, cells were collected and stained with Annexin/PI according to manufacturer’s protocol. Flow cytometry was performed using a BD FACSCanto II with 50,000 events being recorded per sample. Data was analyzed using FlowJo software. Based on forward and side scatter measurements cellular debris was gated out and all experimental data was compensated with single color controls for apoptosis (hydrogen peroxide) and necrosis (heat).

### CRISPR/Cas9 G3BP1 and G3BP2 knockouts

CRISPR guide RNA (gRNA) sequences used in this study were selected and analyzed using the COSMID (CRISPR Off-target Sites with Mismatches, Insertions and Deletions) website (http://crispr.bme.gatech.edu/) to check for any potential off-target sites against the GRCh38 (hg38) genome build and are listed below:

**Table:** 

CRISPR gRNA	Oligonucleotide sequence (5′−3′)
^29^G3BP1 gRNA oligo A	CACCGAAGCCTAGTCCCCTGCTGGT
G3BP1 gRNA oligo B	AAACACCAGCAGGGGACTAGGCTTC
G3BP2 gRNA oligo A	CACCGTGGCCATAAACAGCTTCCTG
G3BP2 gRNA oligo B	AAACCAGGAAGCTGTTTATGGCCAC
non-targeting (nt) gRNA1 oligo A	CACCGGCACTACCAGAGCTAACTCA
non-targeting (nt) gRNA1 oligo B	AAACTGAGTTAGCTCTGGTAGTGCC
non-targeting (nt) gRNA2 oligo A	CACCGCTCATCTATCGCGGTCGTC
non-targeting (nt) gRNA2 oligo B	AAACGACGACCGCGATAGATGAGC

CRISPR gRNA oligonucleotides were annealed and cloned into BbsI-digested lentiCRISPRv2 (Addgene plasmid #52961). Lentivirus was produced by polyethylenimine (PEI)-mediated co-transfection of lentiCRISPR-gRNA with second generation lentiviral vectors pMD2.G and psPAX2 (Addgene plasmid #12259 and #12260, respectively) into human HEK-293T cells. Viral supernatant was collected 48 h post-transfection and used to transduce U251 cells in the presence of 5 μg/ml polybrene (Sigma-Aldrich) for 24 h. To select for viral integration, cells were cultured in the presence of 1 µg/ml puromycin and individual clones were selected and screened for G3BP1 or G3BP2 knockout by western blot.

## Results

### Correlation between mRNAs related to SG dynamics and astrocytoma progression

**Fig. 1:**
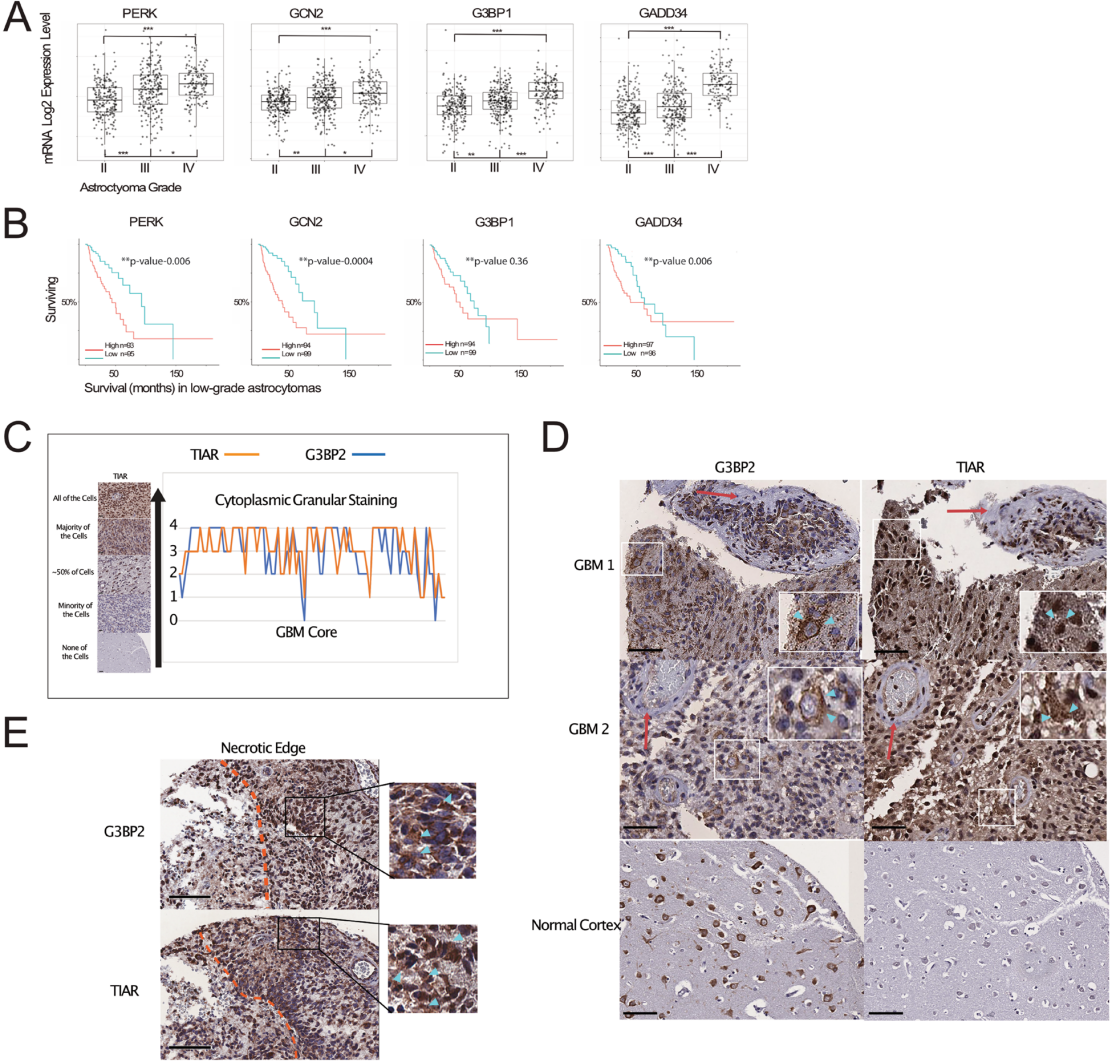
Hypoxic stress responses are activated in low-grade astrocytoma and GBM and correlate to poor outcomes. **A** TCGA database was interrogated with GlioVis for mRNA expression levels of genes involved in activation of the ISR (*PERK*, *GCN2*), in nucleating SGs (*G3BP1*) and terminating the ISR and triggering SG disassembly (*GADD34*) in grade II, III, and IV astrocytoma. Statistical analysis using Tukey’s honest significant difference and performed on GlioVis, ***p < 0.001, **p < 0.01, and *p < 0.05. **B** Kaplan–Meir survival curves for high and low mRNA expression of *PERK*, *GCN2*, *G3BP1*, *GADD34* in low-grade astrocytoma. *p*-values adjusted for multiple comparisons. **C** Graphical representation of IHC cytoplasmic granular scoring of a TMA consisting of 90 GBM cores from 45 patients stained with SG markers G3BP2 and TIAR. Grading was assigned 0–4, no staining, minority of cells, ~50% of cells, majority of cells or all cells (excluding vascular endothelium), respectively. Example of staining grades on left panel. **D** Examples of IHC G3BP2 and TIAR-stained GBM and normal cortex showing cytoplasmic granular staining (blue arrowheads). The vascular endothelium encasing tumor is visible (red arrow) with negative staining of G3BP2 and TIAR. White boxes denote magnified areas. Black line represents 100 μm.  **E** IHC example of GBM core stained for TIAR and G3BP2 demonstrating necrosis (right of orange dotted line) and increased granular staining in the cells adjacent to the necrotic core. Black boxes magnified and granular punctate demonstrated by blue arrow heads. Black line is 100 μm.

TCGA contains RNA-Seq data from hundreds of human GBM samples; this database was interrogated with GlioVis for the mRNA expression levels of two hypoxia-responsive kinases involved in the ISR (*GCN2*, *PERK*), as well as the SG aggregator *G3BP1* and the inducible phosphatase of SG disassembly *GADD34* in Grades II, III, and IV (GBM) astrocytomas. All four genes demonstrated increased expression from low-grade astrocytoma to GBM (Fig. 1A). Expression of these genes did not correlate with survival in GBM, however these genes did predict survival in low-grade astrocytoma with exception of G3BP1 which trended to significance (Fig. 1B). This suggests that the stress response is more active in GBM and in lower-grade astrocytomas with poorer prognosis. In support of an active SG response in GBM we immunohistochemically stained serial sections of a TMA with G3BP2 and TIAR. The majority of GBM samples demonstrated increased cytoplasmic punctate staining in at least some if not most of the core compared to the two normal controls (Fig. 1C, D). Although, the same cells could not be seen between TIAR and G3BP2 the same regions were visible and the correlation between G3BP2 and TIAR was high (Fig. 1C, D). In normal cortex, G3BP2-stained punctae in the cytoplasm of neurons but not the astrocytes and TIAR exhibited no cytoplasmic staining (Fig. 1D). Interestingly, necrotic regions were observed in 36 GBM cores and in 70% of these cores, GBM cells adjacent demonstrated high granular staining (Fig. 1E).

### A cellomics-based screen identified drugs that prevent SG dissolution post-hypoxia in GBM cells

**Fig. 2:**
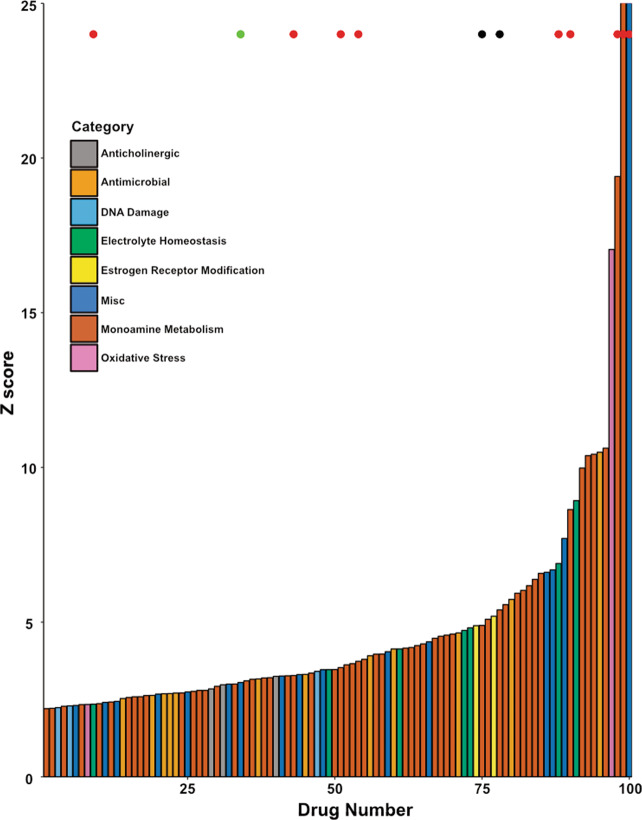
A high-throughput screen identifies drugs showing significant inhibition of SG dissolution 1 h post-hypoxia. U251 human GBM cells were treated with drugs from the Prestwick Chemical Library (1120 drugs, 30 μM each) or vehicle controls for 1 h and exposed to 2 h of hypoxia (~<1% O_2_). Cells were returned to normoxia for 1 h and subsequently fixed and immunostained for TIAR. As a control U251 cells were treated with the library in the absence of hypoxia. SGs were counted and all 1120 drugs were ranked according to their *z*-scores. The top 100 drugs are displayed in order of ascending effect as determined by *z* score (SGs present at 1 h normalized to non-drug controls), and bars are color-coded by drug class. Red dots denote drugs having a high likelihood of being false positives. The green dot represents guanabenz, a known inhibitor of SG dissolution (positive control). Black dots represent the SERMs raloxifene and clomiphene.

The detection of cytoplasmic puncta consistent with SGs in human GBM tissue combined with the observed correlation between astrocytoma progression and high expression of mRNAs related to SG dynamics lead us to further investigate the possibility of altering this pathway pharmacologically. A high-throughput cellomics-based screen of the Prestwick Chemical Library was conducted to identify drugs that impacted SG dissolution post-hypoxic stress in U251 human GBM cells. The top 100 drugs with the highest *z*-score changes were identified, plotted, and colored by drug class (Fig. 2; Supplementary Table 1). The majority of the top 100 drugs (55%) belonged to classes of drugs interfering with monoamine metabolism, however no obvious trends were apparent regarding specific drug classes. Nine drugs induced SGs in normoxia and were removed from further consideration (red circles Fig. 2). Guanabenz is a known drug which sustains eIF2α phosphorylation and delays SG dissolution^30^; this drug served as a positive control in our screen and is denoted by the green dot in Fig. 2. Candidate molecules with known links to GBM, hypoxia, oxidative stress, or SGs were retained for further analysis. Notably, the SERMs raloxifene and clomiphene, which are auxiliary therapeutic modalities in GBM^31,32^, both delayed SG dissolution in U251 cells post-hypoxia (black circles Fig. 2).

### Raloxifene delays dissolution of hypoxia-induced SGs in GBM cells

**Fig. 3:**
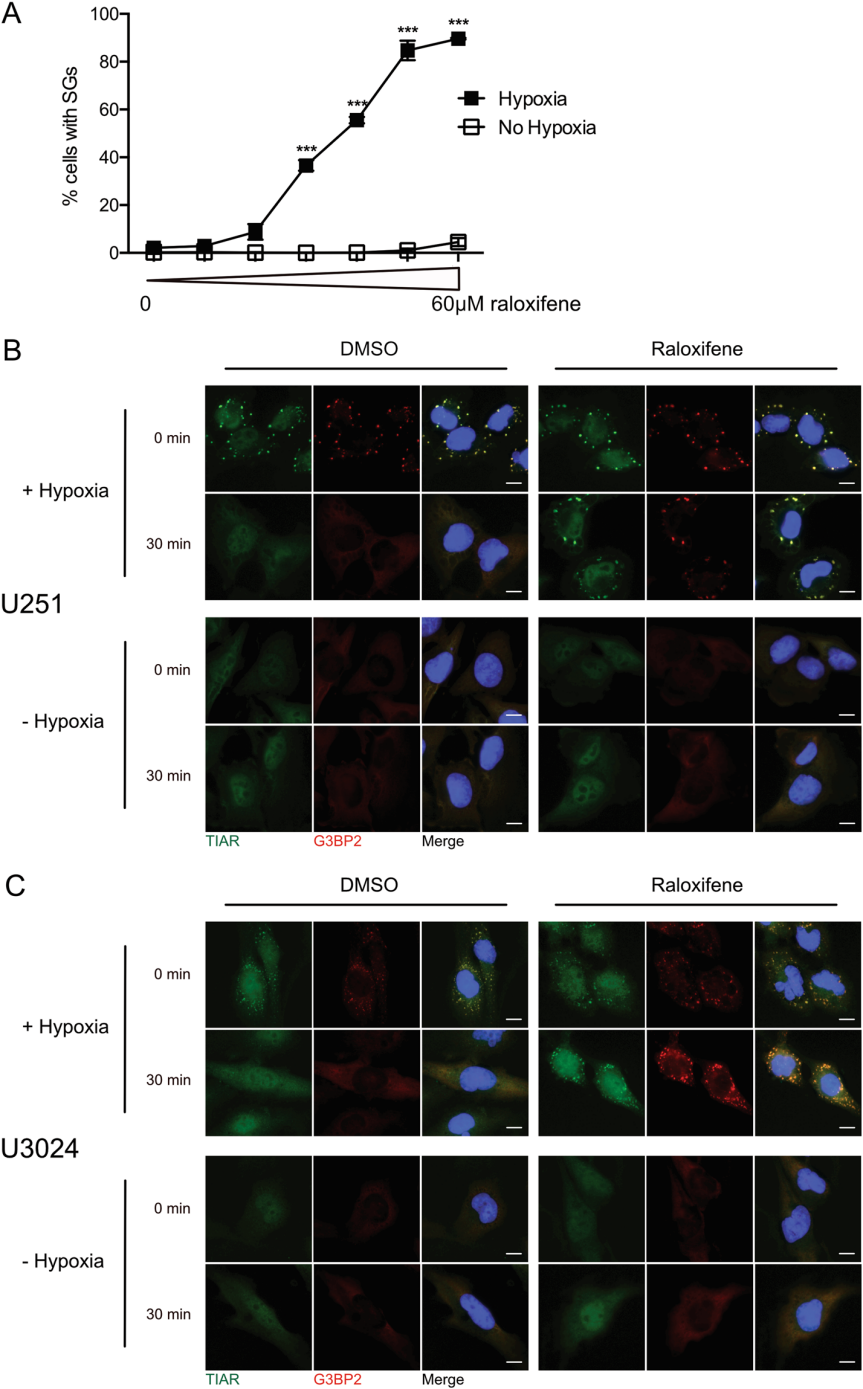
Raloxifene inhibits dissolution of hypoxia-induced SGs in glioblastoma cells and does so in a dose-dependent manner. **A** Raloxifene dose response curve. U251 glioblastoma cells were treated with raloxifene at doses ranging from 0 to 60 μM for 1 h prior to a 2 h incubation ± hypoxia (<1% O_2_). Cells were allowed to recover for 1 h in normoxia and then fixed and stained for SG markers TIAR and FMRP. Percentage of cells with SGs were counted manually; 10 fields of view ~50 cells per field. Data is presented as the mean of triplicates ± SEM, unpaired *t*-test ***p < 0.001. **B** Immortalized GBM U251 cells and **C** primary GBM U3024 cells were treated with 40 μM raloxifene or vehicle control (DMSO) for 1 h followed by a 2 h (U251) or 1 h (U3024) incubation ± hypoxia (<1% O_2_). Cells were then fixed immediately (0 min) or allowed to recover (30 min) in normoxia before being fixed and stained for SG markers TIAR (green) and G3BP2 (red). DNA was counterstained with DAPI (blue). Scale bars = 10 microns.

As secondary validation, we conducted a dose response curve of raloxifene’s effect on hypoxia-induced SGs by treating U251 cells with escalating doses of raloxifene (0–60 μM). Doses as low as 20 μM prevented SG dissolution post-hypoxia, while maximal suppression of SG dissolution was observed at 50–60 μM (Fig. 3A). Conversely, raloxifene did not induce SGs at any dose in U251 cells cultured in normoxic conditions (Fig. 3A). A dose of 40 μM was selected for subsequent testing as it was the median dose that elicited a significant SG response.

Sustained SGs in the presence of raloxifene was visually confirmed by dual TIAR and G3BP2 immunofluorescence staining in both the immortalized GBM cell line U251, in addition to a primary GBM cell line U3024 (top panels Fig. 3B, C). Raloxifene alone did not induce SGs in either cell line during normoxia (bottom panels Fig. 3B, C).

**Fig. 4:**
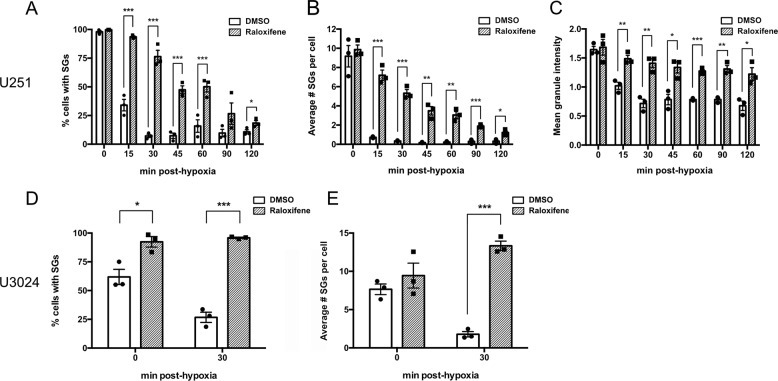
Raloxifene delays SG dissolution for up to 2 h post-hypoxia. Quantitative analysis of SGs. Immortalized U251 **A**–**C** and primary U3024 GBM cells **D** and **E** were treated with 40 μM raloxifene or vehicle control (DMSO) for 1 h before being subjected to either 2 h (U251) or 1 h (U3024) of hypoxia (<1% O_2_). Cells were fixed at various times post-hypoxia and stained for SG markers TIAR and G3BP2. Cells were also stained with wheat germ agglutinin to denote cellular membranes and DAPI to identify nuclei. Percentage of cells with SGs and average number of SGs per cell (in those cells containing SGs) or SG intensity were then quantified from correlative TIAR and G3BP2 staining using an automated image analysis pipeline in CellProfiler. Data is presented as the mean of triplicates ± SEM, unpaired *t*-test. *p < 0.05; **p < 0.01***; and *p* < 0.001.

To describe our findings in a more objective manner, we developed an automated image analysis pipeline in CellProfiler (see “Materials and methods” section) to quantify the percentage of cells containing SGs, SG number per cell, and SG intensity (used as a correlative measurement for SG size). Immediately post-hypoxia, U251 cells exhibited a robust SG response irrespective of the presence of raloxifene, with no significant difference in percentage of cells exhibiting SGs, SGs/cell, or SG intensity (Fig. 4A–C). Complete SG dissolution occurred within 15–30 min post-hypoxia in control cells; however, the rate of SG dissolution following hypoxia was significantly slower in cells pre-treated with raloxifene (Fig. 4A, B). These results were further confirmed in the U3024 primary GBM cell line. Similar to U251s, raloxifene pre-treatment in these cells leads to sustained SGs (Fig. 4D, E); however, the initial percentage of cells with SGs is significantly higher with raloxifene pre-treatment (92% and 65%, respectively, Fig. 4D).

### Raloxifene inhibits resumption of bulk translation post-hypoxia

**Fig. 5:**
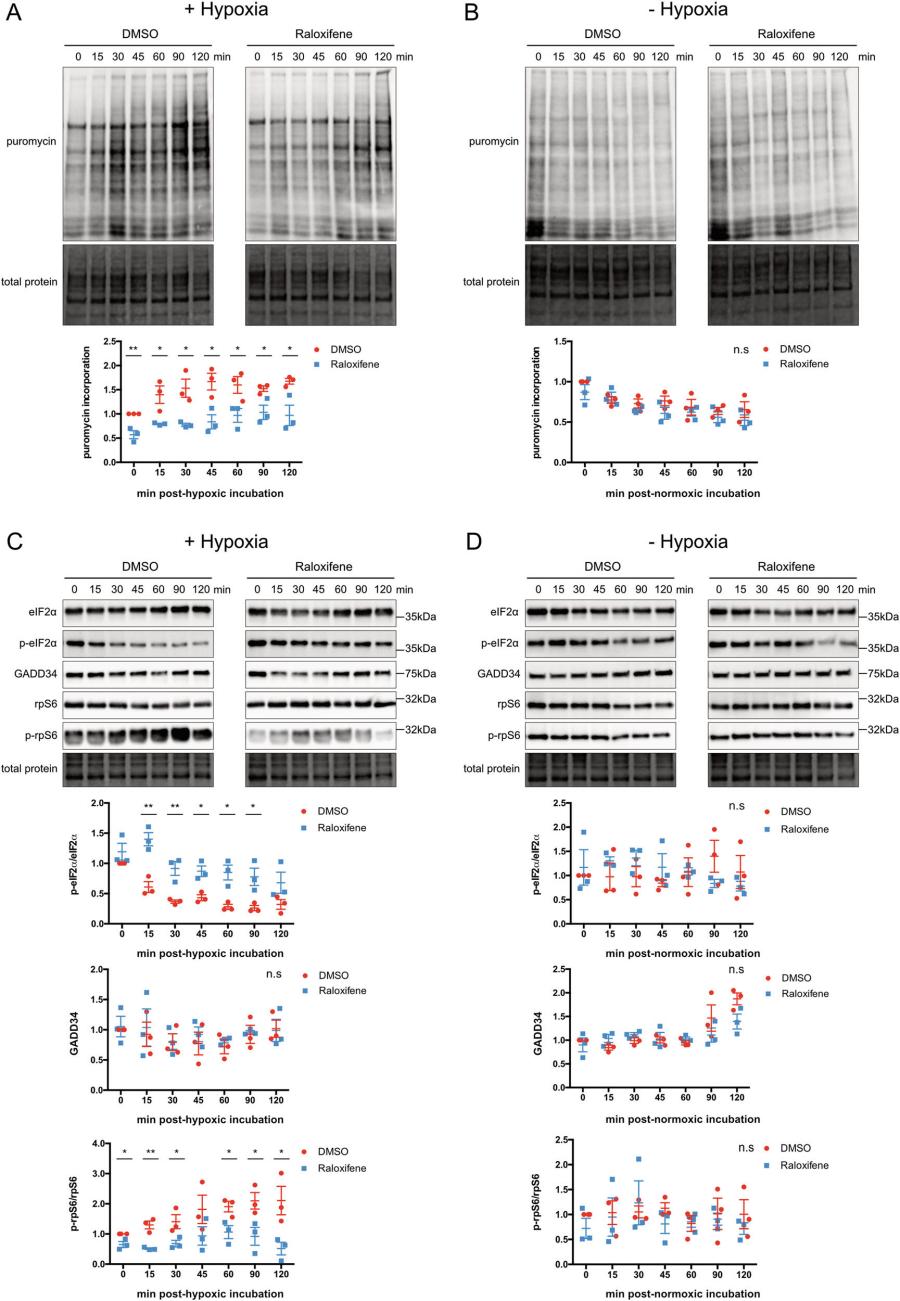
Raloxifene delays resumption of protein translation post-hypoxia in correlation with increased eIF2α phosphorylation and decreased mTOR signaling. **A** and **B** Puromycin incorporation assay. U251 cells were treated with 40 μM raloxifene or DMSO vehicle control for 1 h prior to 2 h hypoxic **A** or non-hypoxic **B** incubation. At various times post-hypoxic or normoxic incubation cells were treated with 10 μg/mL puromycin for 10 min and puromycin incorporation into nascent proteins was detected by anti-puromycin blot. Blots were normalized to total lane protein, and represented as ratios with DMSO time 0 normalized to 1 (bottom quantification panels). Data is presented as the mean of triplicates ± SEM, unpaired *t*-test *p < 0.05; **p < 0.01. **C** and **D** U251 cells were treated as in **A** but without addition of puromycin. Cell lysates were probed for eIF2α, phospho-eIF2α, GADD34, rpS6, and phospho-rpS6 at various times post-hypoxic **C** or normoxic **D** incubation. All blots were normalized to total lane protein with DMSO time 0 set to 1 (bottom quantification panels). The level of eIF2α phosphorylation is presented as the ratio of p-eIF2α to total eIF2α (bottom quantification panels). The level of rpS6 phosphorylation is similarly presented. Data is presented as the mean of triplicates ± SEM, unpaired *t*-test, and false discovery rate of 1%. *p < 0.05; **p < 0.01.

SG formation is traditionally coupled with stress-induced translational silencing, therefore we wanted to determine if raloxifene-induced granulostasis exhibited differences in global protein translation compared to a normal SG response. To measure global protein synthesis levels, raloxifene and vehicle-treated cells were pulsed with puromycin at various times post-hypoxia. Puromycin incorporation into nascent polypeptide chains was then visualized by western blot, quantified, and used as an indicator of overall protein synthesis. A steady increase in protein translation can be observed in control cells post-hypoxia, concomitant with loss of SGs (Figs. 5A and 4). However, immediately post-hypoxia, protein translation was significantly decreased in cells pre-treated with raloxifene (Fig. 5A) and remained low for the duration of the 2 h stress recovery period, correlating with the persistence of SGs in these cells (Fig. 4). Importantly this was not due to raloxifene itself impacting protein translation, as raloxifene-treated cells that were maintained in normoxia for the same duration of time displayed no difference in puromycin incorporation relative to vehicle control cells (Fig. 5B). See Supplementary Fig. 1 for whole western blot images.

Canonical SG presence requires phosphorylation of the α subunit of eIF2α, an event which blocks translation initiation^33,34^. In correlation with the observed decrease in global protein translation, raloxifene treatment resulted in sustained eIF2α phosphorylation post-hypoxia. This was in direct contrast to control cells in which eIF2α phosphorylation diminished with increasing global translation and loss of SGs (Fig. 5C). Raloxifene alone was not responsible for this phosphorylation event as raloxifene and vehicle-treated cells that were not made hypoxic showed no difference in phospho-eIF2α (Fig. 5D).

Dephosphorylation of phospho-eIF2α is accomplished by stress-induced cellular upregulation of the phosphatase GADD34. We initially suspected that failed upregulation of GADD34 could explain the sustained phospho-eIF2α observed in raloxifene-treated cells. Surprisingly, GADD34 protein levels do not change between raloxifene and control cells post-hypoxia (Fig. 5C) suggesting granulostasis, and that sustained eIF2α phosphorylation and concomitant SGs are occurring through a different mechanism. See Supplementary Figs. 1 and 2 for whole western blot images.

### Raloxifene prevents re-activation of mTOR signaling post-hypoxia

Mammalian target of rapamycin (mTOR), is a key GBM oncogene that phosphorylates a wide variety of substrates involved in stress recovery and cancer pathogenesis. During stress, mTOR function is diminished to conserve cellular energy^35,36^. We previously demonstrated that mTOR effectors are contained in SGs^37^ and therefore postulated that mTOR activity would be low in U251 cells with sustained SGs. To ascertain the status of mTOR signaling in our model, we examined the phosphorylation level of the mTOR effector rpS6 in raloxifene-treated cells post-hypoxia. rpS6 phosphorylation was decreased up to 2 h post-hypoxia in raloxifene-treated cells relative to control cells (Fig. 5C) in correlation with sustained SGs. No change was detected in phospho-rpS6 levels between raloxifene and control cells that were maintained in normoxia (Fig. 5D). See Supplementary Fig. 2 for whole western blot images.

### The combination of raloxifene and hypoxia results in GBM cell death and in autophagic inhibition similar to CQ

**Fig. 6:**
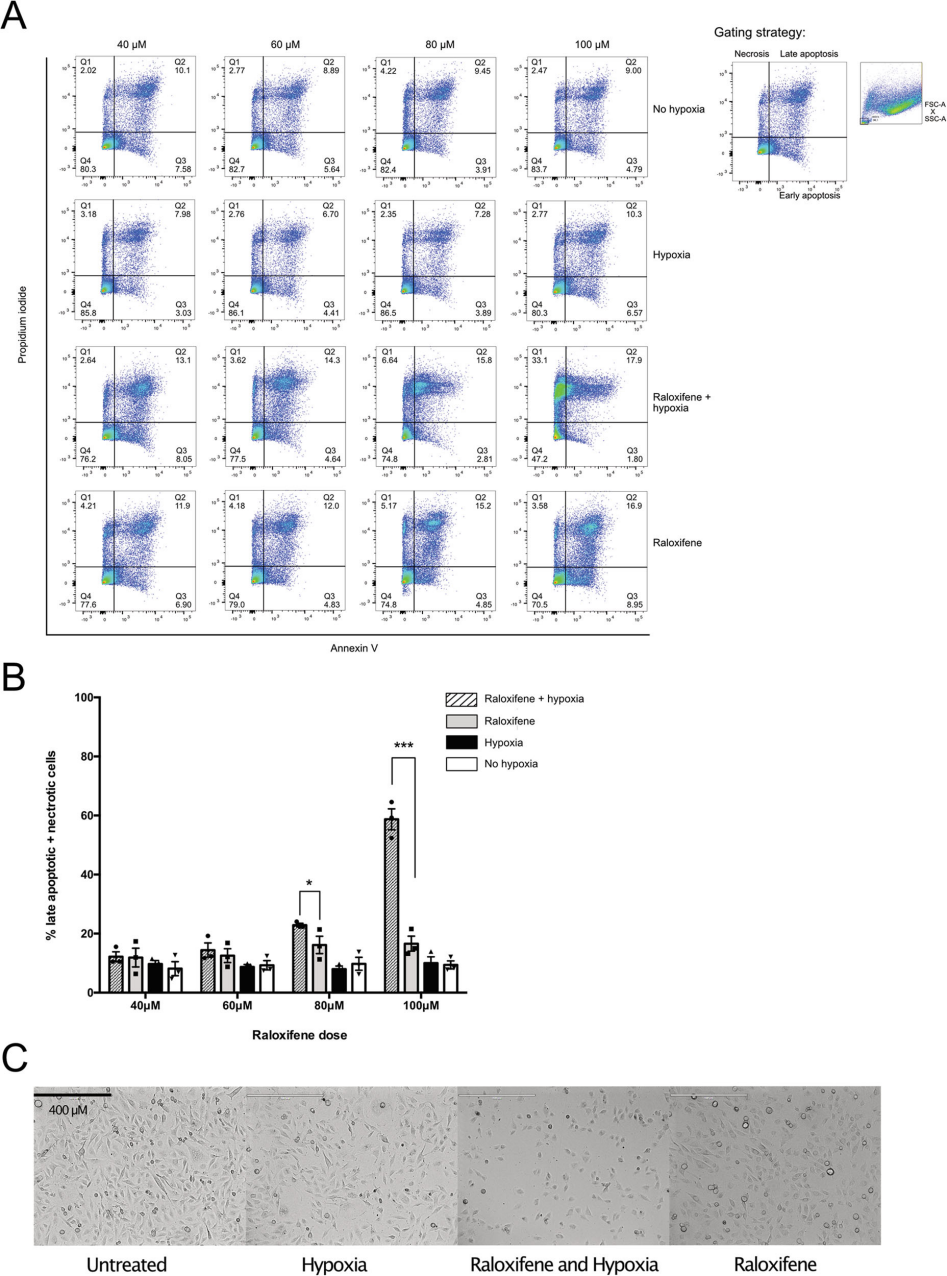
Raloxifene results in increased cell death with hypoxia. **A** U251 cells were treated with raloxifene (or equivalent vehicle control) at doses ranging from 40 to 100 μM for 1 h prior to a 2 h incubation ± hypoxia (<1% O_2_). Cells were allowed to recover for 2 h in normoxia, labeled live with annexin V and PI and analyzed by flow cytometry. Cellular debris based on forward and side scatter was excluded from analysis (see right inset). Cells in the lower left (Q4) quadrant (annexin and PI negative) were classified as living, the lower right (Q3) quadrant (annexin positive) as early apoptotic, the upper right (Q2) quadrant (annexin and PI positive) as late apoptotic and cells in the upper left (Q1) quadrant (PI positive) were considered necrotic (see right inset). Representative dot plots display 50,000 events of annexin V versus PI for each concentration and condition. **B** Percentage of late apoptotic and necrotic populations were combined and presented graphically as the mean of triplicates ± SEM, unpaired *t*-test and false discovery rate of 1%. *p < 0.05; **p < 0.01; ***p < 0.001. **C** Brightfield microscopy of vehicle control and raloxifene-treated (40 µM) cells 2 h post ± hypoxia.

The addition of raloxifene prior to hypoxic stress appears to prolong stress recovery even after cells are returned to normoxia. Without the ability to resolve stress, we postulated that cell viability would be adversely impacted. Cell death was evaluated by determining levels of early and late apoptosis/necrosis with AnnexinV/PI staining. Increasing doses of raloxifene in combination with hypoxia displayed a significant shift in the number of cells undergoing late apoptosis/necrosis (Fig. 6A, B). Importantly while the amount of apoptosis occurring in raloxifene-treated cells was higher than vehicle control or hypoxia alone, it did not exhibit the shift towards late apoptosis/necrosis seen with the combination of raloxifene and hypoxia (Fig. 6A).

**Fig. 7:**
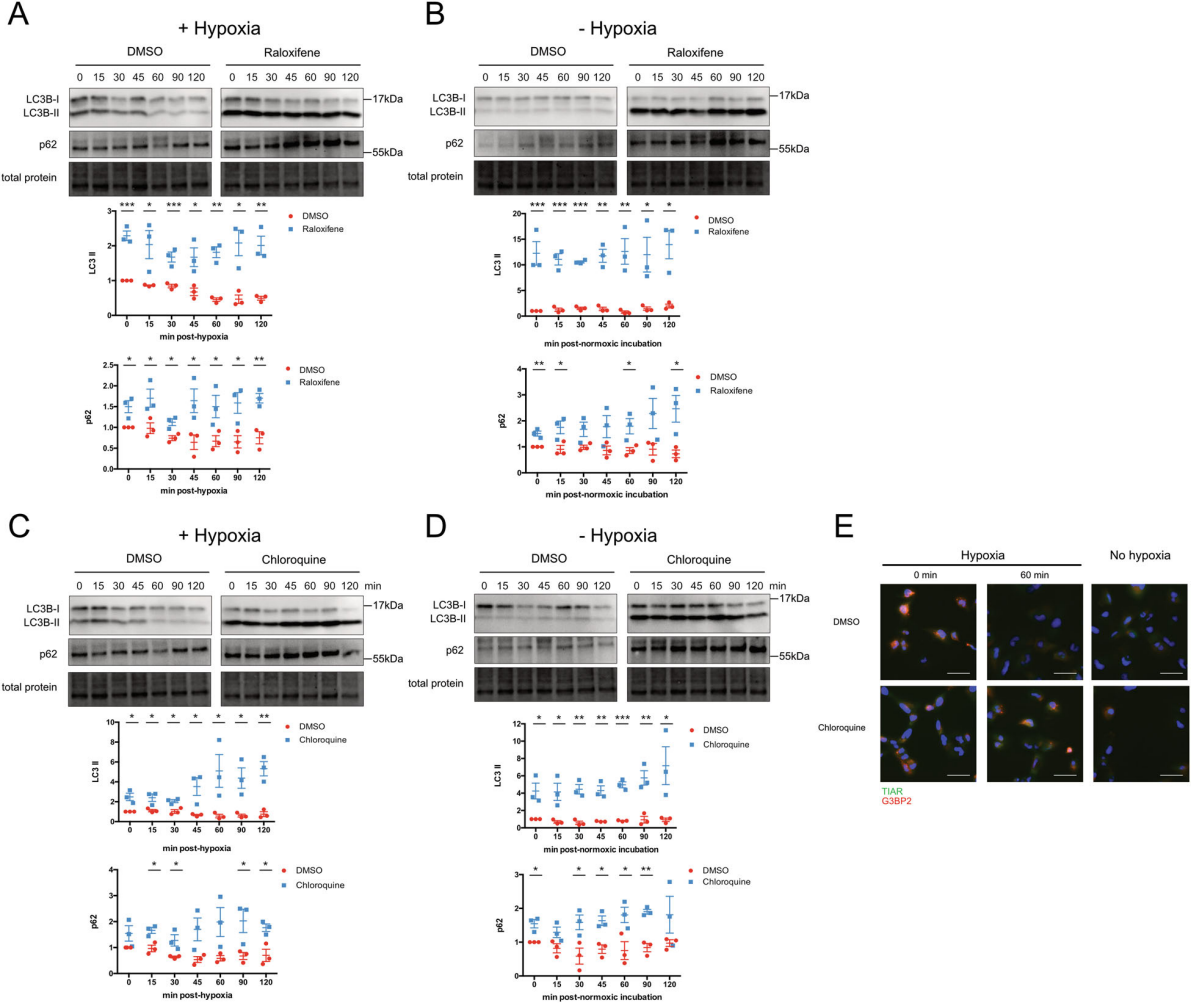
Raloxifene causes a block in autophagy similar to the known autophagy inhibitor chloroquine. Cells were treated with 40 μM raloxifene **A** and **B** or 40 μM chloroquine **C** and **D** or DMSO vehicle control for 1 h prior to a 2 h incubation ± hypoxia (<1% O_2_). At various times post-hypoxic or normoxic incubation cells were lysed and lysates were probed for LC3B and p62. All blots were normalized to total lane protein with DMSO time 0 set to 1 (bottom quantification panels). **E** CQ or vehicle control treated cells were fixed and stained for TIAR and G3BP2 immediately (0 min) or 30 min post-hypoxic or normoxic incubation. Scale bars = 50 microns. Data is presented as the mean of triplicates ± SEM, unpaired *t*-test and false discovery rate of 1%. *p < 0.05; **p < 0.01; ***p < 0.001.

It is known that autophagy is required for clearance of retained SGs and there is cross-talk between apoptosis, necrosis, and autophagy^38^, therefore we asked whether defects in autophagy could account for retained SGs. LC3-II is a protein required during the expansion/elongation phase of autophagy. LC3-II levels also increase when the fusion of autophagosomes with lysosomes is inhibited, such as by treatment with the known lysosomal inhibitor CQ^39^. Raloxifene treatment with or without hypoxia lead to significantly increased LC3-II levels (Fig. 7A, B), comparable to CQ (Fig. 7C, D). Similarly, increases in p62 are associated with a block in autophagy and were also observed with raloxifene and CQ treatment (Fig. 7A–D). Interestingly the combination of hypoxia and CQ also resulted in sustained SGs (Fig. 7E). See Supplementary Fig. 5 for whole western blot images.

As raloxifene is a SERM, we initially attempted to determine whether raloxifene-induced granulostasis and cell death was via antagonistic or agonistic effects at the estrogen receptor. No concentration of β-estradiol could mimic or rescue granulostatic effect (Supplementary Fig. 3). We also attempted to rescue SG stasis with the addition of the anti-oxidant pyruvate, however this failed to prevent granulostais suggesting raloxifene is not acting as a pro-oxidant (Supplementary Fig. 4)^40,41^.

### G3BP1 knock-out partially restores SG dissolution in raloxifene-treated cells

**Fig. 8:**
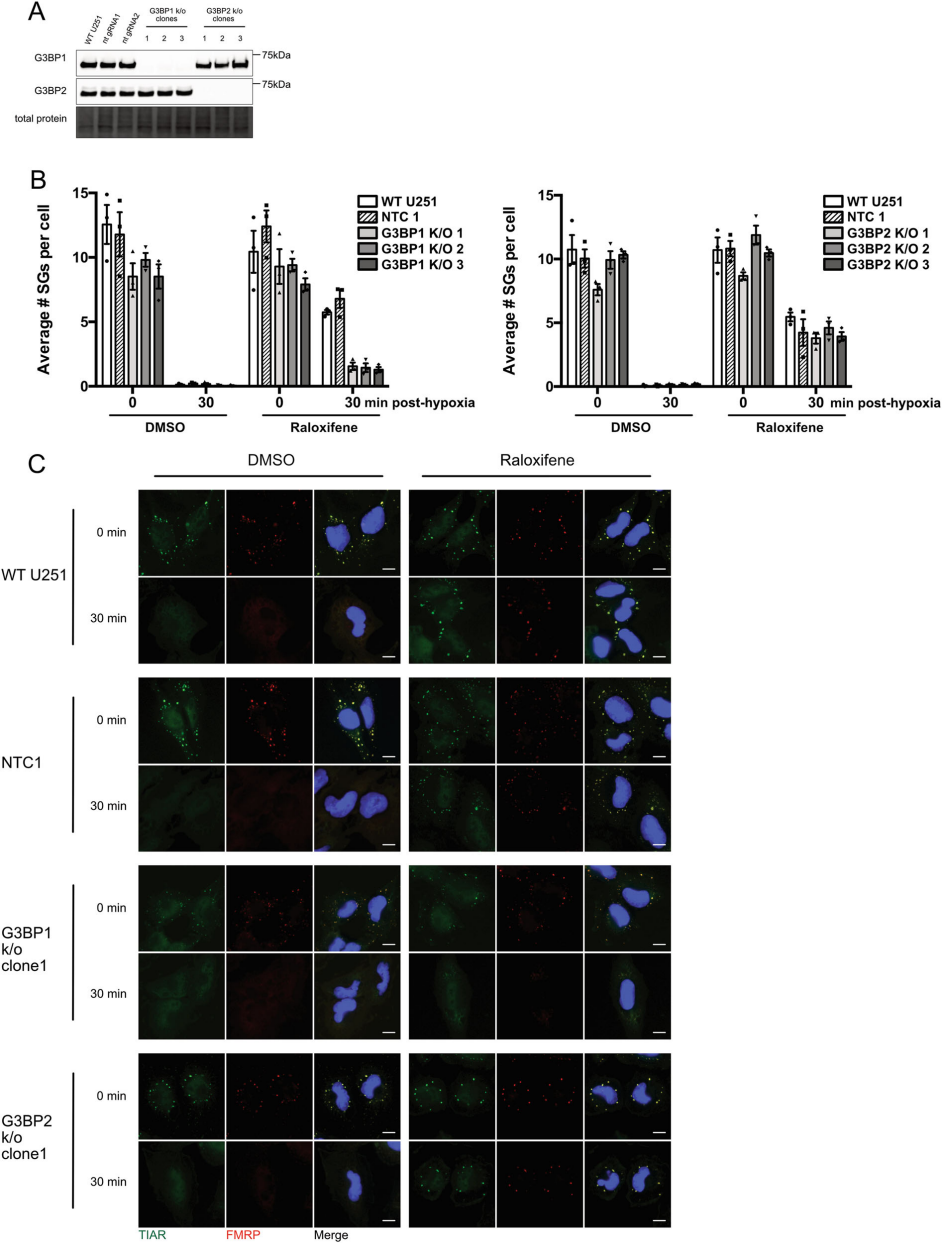
G3BP1 knockout partially reverses raloxifene-induced delay of SG dissolution. **A** U251 cell lines stably expressing CRISPR gRNAs targeting G3BP1 or G3BP2 along with non-targeting (nt) gRNAs were established using a lentiviral-based system. **B** Untransduced (WT) U251 and nt gRNA control (NTC) cells along with G3BP1 and G3BP2 knockout clones were treated with 40 μM raloxifene or DMSO vehicle control prior to 2 h of hypoxia (<1% O_2_). Cells were fixed either immediately (0 min) or 30 min post-hypoxia. Cells were stained for CellProfiler analysis as previously described, and average number of SGs per cell (in those cells containing SGs) were quantified from correlative TIAR and FMRP staining in CellProfiler. Data is presented as the mean of triplicates ± SEM. **C** Representative immunofluorescence images. Cells were stained for SG markers TIAR (green) and FMRP (red), and DNA was counterstained with DAPI (blue) immediately (0 min) or 30 min post-hypoxia. Scale bars = 10 microns.

A recent publication suggested G3BP1 may be required for SG dissolution during hyperactive stress^42^. G3BP1 and G3BP2 are SG aggregating proteins that are critically important for SG assembly and loss of both are required to inhibit SG formation in response to several stressors, but not hypoxia^29,43^. We generated U251 G3BP1 and G3BP2 null cell lines using a CRISPR lentiviral delivery system (Fig. 8A). We pre-treated G3BP1 null, G3BP2 null, wild-type U251, and a non-targeting gRNA control (NTC) cell line with raloxifene or vehicle control prior to hypoxia. Interestingly, while G3BP2 null cells behaved largely like wild-type and NTC cells, the loss of G3BP1 partially restored SG dissolution in raloxifene-treated cells post-hypoxia (Fig. 8B, C).

## Discussion

GBM is a highly aggressive tumor with a high rate of growth and invasiveness into the normal brain. These malignant tumors exist in a hostile microenvironment due to cells outstripping their local nutrient supplies (oxygen, glucose, amino acids) and the addition of chemotherapeutic-induced and radiation-induced stress. Despite these stressors, GBM cells continue to thrive and overcome treatment, resulting in a life expectancy of 14–16 months post-diagnosis^1^. This implies GBM cells have the ability to adapt to microenvironmental stressors. Emerging evidence suggests that stress adaptation happens through proteomic reprogramming at the translational level^18,19^. One mechanism of stress reprogramming is the sequestration of mRNAs and proteins into SGs.

Mining hundreds of RNA-Seq samples from human GBMs in TCGA databases demonstrates that mRNAs involved in the ISR and both SG formation and dissolution are upregulated in GBM compared to lower-grade, less aggressive astrocytomas (Fig. 1). Although mRNA expression did not predict survival in human patients with GBM, these mRNAs did predict poor survival when upregulated in the less aggressive, lower grade astrocytomas. It is known that all lower grade astrocytomas progress to their more malignant counterparts over time, in fact patients do not die from their low-grade astrocytoma but from malignant transformation. We hypothesize that lower-grade astrocytomas that demonstrate upregulation of these stress response genes may be exhibiting micro-areas of increased metabolic stress (nutrient starvation, hypoxia, etc.) that mark the beginning of malignant transformation, hence high expression relates to poor survival. Perhaps, upregulation of these stress-response genes can be used as markers of progression. Although provocative, this would need to be confirmed in a prospective manner with pathological correlates and protein data.

Our previous work demonstrated that SGs could form in GBM stem cells and culture in vitro^37^. Here we have demonstrated for the first time the presence of cytoplasmic aggregates reminiscent of SGs in operative human GBM tissues. Although normal cortical neurons displayed punctae of G3BP2 the background astrocytic cells did not, G3BP is known to form punctae in neuronal cell bodies^44^. Intriguingly cells near areas of necrosis demonstrated more intracellular TIAR and G3BP2 aggregates by visual inspection (Fig. 1). These areas of necrosis and the “pseudo-pallisading” adjacent cells are known to be hypoxic^45^. Although a promising indication that SGs exist in GBM and may be important in adaptation to environmental stressors, current technical limitations prevents us from fully characterizing these granules and confirming their identity as SGs (that they also contain RNA).

Given that TCGA data demonstrates that both the kinases that result in SG aggregation and the phosphatase that triggers SG dissolution are upregulated in GBM, we postulated that the cycling of SGs may be important in GBM cellular adaptation. Therefore, we hypothesized that pharmacologically impairing SG dissolution and restoration of translation would affect cellular homeostasis. This is consistent with observations in other disease conditions, such as viral infection and neurodegenerative disease where the dissolution and cycling of SGs are important^46–49^. To explore our hypothesis, we screened chemical compounds that resulted in granulostasis in U251 GBM cells after recovery from hypoxia-induced stress. Hypoxia was chosen as a stressor as GBM experiences high degrees of hypoxia which has been shown to promote tumor aggressiveness, stemness, invasion, and resistance to therapy^50–52^. Our screen identified 100 compounds with granulostasis and *z*-scores of >2.2 when compared to our negative controls (Fig. 2; Supplementary Table 1). We secondarily validated the drug raloxifene as having a dose-dependant ability to cause granulostasis in both an immortalized and a primary GBM stem cell line (Figs. 3 and 4). This was interesting to us as SERMs have been used as salvage chemotherapy with some effect in GBM and have been postulated to be radiation and chemotherapy sensitizers^53,54^

Typically, SG formation results in high levels of phospho-eIF2α, translational repression^55^, and suppression of mTOR activation^55–58^. We have demonstrated that following hypoxia, control cells dissolve SGs, dephosphorylate eIF2α, and restore translation and mTOR activity within 15 min. Raloxifene treatment delays these processes at a rate coincident with SG dissolution beginning at around 1.5–2 h following release from hypoxia (Fig. 5A, C). mTOR is a key oncogene that drives GBM pathogenesis and is constitutively active in U251 cells due to an upstream frameshift mutation in the tumor suppressor PTEN^59^. It is significant that we were able to alter restoration of homeostasis and mTOR activity after hypoxia by delaying SG dissolution, albeit for a short-interval of time. We believe this lack of mTOR activity is due to translational silencing during granulostasis, however a direct inhibition of mTOR by raloxifene has not been ruled out and these experiments could be repeated in the presence of an mTOR inhibitor such as torin. However, if we could harness this dampening effect of mTOR for longer, this alone would be of significant clinical value as mTOR is a major GBM oncogene. This could be accomplished by either altering raloxifene for a more prolonged effect or by changing delivery methods (sustained release, decreasing dosing intervals).

Since the stress-induced phosphatase GADD34 dephosphorylates phospho-eIF2α, we questioned whether granulostasis resulted from failed GADD34 induction. We found no changes in GADD34 levels between hypoxia alone or raloxifene/hypoxia (Fig. 5C). Whether raloxifene impairs GADD34’s ability to associate with phospho-eIF2α remains to be tested but is a possibility.

Cycling of SGs is important to cell survival. If SGs are indeed a targetable pathway for GBM treatment, then it must follow that raloxifene is able to increase astrocytoma cell death. Consistent with this, increasing doses of raloxifene when combined with hypoxia lead to increasing granulostasis and a shift towards late apoptosis and necrosis (Fig. 6). We demonstrated that raloxifene treatment caused a block in late autophagy, as indicated by the accumulation of LC3-II over time of treatment (Fig. 7A, B). The LC3-II accumulation we observed was nearly identical to cells treated with the known autophagy blocker, CQ (Fig. 7C, D). We also observed that subjecting untreated cells to hypoxic conditions increased LC3-II levels, consistent with previous reports, and that these levels returned to baseline upon return to normoxia.^60,61^ However, treatment with raloxifene post-hypoxia caused LC3-II and p62 steady-state levels to remain high, even upon restoration of normoxia. These observations are consistent with the following model: hypoxia increases autophagic flux but a late stage blockage in flux induced by CQ or raloxifene causes a failure of lysosomal degradation, and a shift towards necrosis. Another interesting observation was that raloxifene treatment alone did not result in granulostasis, alter translation or induce phospho-eIF2α and phospho-rpS6 levels compared to vehicle controls, suggesting two important interpretations. The first is that the stressful event of hypoxia exposure is required to initiate a sequence of events (eIF2α phosphorylation, SG formation, and enhanced autophagic flux) aimed at promoting cell survival. When raloxifene is added to this ‘stressed’ system, the cell fails to survive. The second is that because hypoxia and raloxifene work synergistically to induce cell death, raloxifene treatment is less likely to be toxic to normoxic/healthy tissues. Provocatively, SERMs prevent oxidative stress in the brains of ovariectomized rats^62^ and have been implicated as a neuro-protective agent in aging of the brain^63^. This potential dichotomy of raloxifene will need to be further explored utilizing humanized mouse models of GBM.

Because CQ is a known inhibitor of autophagic lysosomal degradation^39^, we wanted to determine if CQ worked similarly to raloxifene. CQ exhibited a similar phenomenon; hypoxia-induced SGs were retained after CQ as observed in raloxifene-treated cells (Fig. 7E). Previous work has shown that CQ can trigger cell death independent of apoptosis in GBM due to accumulation of autophagasomes^39,64,65^. Typically, SGs are cleared by the PQC and the HSP8–BAG3–HSP70 complex^14^ but as granulostasis persists SGs are cleared by autophagy^16,38^. In keeping with this, a block in late autophagy can result in SG accumulation as we observed in both raloxifene/hypoxia and CQ/hypoxia-treated and cells (Figs. 4, 7D). Taken together, these data suggest that raloxifene is blocking late autophagy at a stage similar to CQ, resulting in unresolved autophagosome accumulation and eventual shift to necrosis. In future studies, we will further interrogate this mechanisms by staining for autophagosomes, knocking down canonical autophagy regulators (ATG5) and using chemicals that inhibit different stages of autophagic flux (torin, wortmannin, 3-MA).

We had previously generated G3BP1 and G3BP2 knockout U251 cell lines. These cells still form SGs in the presence of hypoxia as both G3BP1/2 need to be lost to prevent hypoxia-induced SG formation, similar to what has been described previously^29^. Interestingly, loss of G3BP1 but not G3BP2 resulted in partial rescue of raloxifene/hypoxia-induced granulostasis (Fig. 8). This is intriguing as acetylation of G3BP1 at lysine-376 regulated by histone deacetylase-6 (HDAC6) has been directly linked to SG dissolution during hyperactive stress^42^. One possibility is that raloxifene alters acetylation of G3BP1 resulting in failure of SG dissolution and that loss of G3BP1 (perhaps G3BP1 acts a dominant negative) allows G3BP2 to take more of an active role in dissolution. Interestingly HDAC6 and autophagy are linked^66,67^, further supporting that G3BP1 and HDAC6 have a role in the SG stasis induced by raloxifene/hypoxia. These experiments are on-going in our laboratory.

SERMs have been shown to be effective against GBM in vitro as a chemo- and radiation sensitizer, and to have select activity in a subset of GBM patients. Although clinical trials of SERMs alone have not been effective, one wonders whether SERMs such as raloxifene may be more effective if combined with a chemotherapeutic that induces SGs (such as bortezomib^68^, vinca alkaloids, or 5-fluorouracil) or other late blockers of autophagy. In addition, it is important to recognize that raloxifene may not be the best compound to exert SG stasis, but it works as a proof-of-concept that altering SG dynamics impairs cellular homeostasis after stress. Our screen results (100 potential candidates) demonstrates that SG cycling is a druggable pathway and other candidates should be investigated alone or in combination for a more robust effect on GBM cell viability. We have provided evidence that this pathway deserves further exploration as a viable therapeutic target.

## Supplementary information

Supplemental Table 1

Supplemental Figure 1

Supplemental Figure 2

Supplemental Figure 3

Supplemental Figure 4

Supplemental Figure 5

Supplemental Figure Legends

Supplemental Materials and Methods
