# Supplementary material for: Raloxifene prevents stress granule dissolution, impairs translational control and promotes cell death during hypoxia in glioblastoma cells
Source: Cell Death Dis. 2020 Nov 17;11(11):989. doi: 10.1038/s41419-020-03159-5 (PMC7673037; doi:10.1038/s41419-020-03159-5)
Supplement: Supplementary file 1 — Supplemental Table 1 [file 41419_2020_3159_MOESM1_ESM.docx]

**Table S1. Top 100 screened drugs inhibiting SG dissolution.**

| **Drug** | **Drug Class** | **Z Score** |
| --- | --- | --- |
| **Chelidonine (+)** | Monoamine Metabolism | 47.59765484 |
| **Scoulerine** | Monoamine Metabolism | 40.01073732 |
| **Piperlongumine** | Oxidative Stress | 19.40302893 |
| Lobeline alpha (-) hydrochoride | Monoamine Metabolism | 17.04493294 |
| Benzethonium chloride | Antimicrobial | 10.62192853 |
| Metergoline | Monoamine Metabolism | 10.4904695 |
| Nortriptyline hydrochloride | Monoamine Metabolism | 10.42626503 |
| Lobelanidine hydrochloride | Monoamine Metabolism | 10.38074863 |
| Benzamil hydrochloride | Electrolyte Homeostasis | 9.980604543 |
| Desipramine hydrochloride | Monoamine Metabolism | 8.927833286 |
| **Eburnamonine (-)** | Misc | 8.637808122 |
| Bepridil hydrochloride | Electrolyte Homeostasis | 7.704900593 |
| **Berberine chloride** | Misc | 6.894867611 |
| Dimethisoquin hydrochloride | Misc | 6.689815786 |
| Ifenprodil tartrate | Monoamine Metabolism | 6.611121205 |
| Promazine hydrochloride | Monoamine Metabolism | 6.576654152 |
| Methoxy-6-harmalan | Monoamine Metabolism | 6.382238049 |
| Hydrastine hydrochloride | Monoamine Metabolism | 6.177186224 |
| Homochlorcyclizine dihydrochloride | Monoamine Metabolism | 6.026542715 |
| Methyl benzethonium chloride | Antimicrobial | 5.935330165 |
| Thiethylperazine malate | Monoamine Metabolism | 5.734660553 |
| Clemastine fumarate | Monoamine Metabolism | 5.565033382 |
| **Clomiphene citrate (Z,E)** | Estrogen Receptor Modification | 5.394916618 |
| Dobutamine hydrochloride | Monoamine Metabolism | 5.191094503 |
| Methotrimeprazine maleat salt | Monoamine Metabolism | 5.093076953 |
| **Raloxifene hydrochloride** | Estrogen Receptor Modification | 4.894554443 |
| Lidoflazine | Electrolyte Homeostasis | 4.885361329 |
| Nicardipine hydrochloride | Electrolyte Homeostasis | 4.817420624 |
| Parbendazole | Antimicrobial | 4.733216072 |
| Trimipramine maleate salt | Monoamine Metabolism | 4.651681997 |
| Chlorprothixene hydrochloride | Monoamine Metabolism | 4.613598509 |
| Methiothepin maleate | Monoamine Metabolism | 4.579628156 |
| Protriptyline hydrochloride | Monoamine Metabolism | 4.544257097 |
| Thonzonium bromide | Misc | 4.479818592 |
| Doxepin hydrochloride | Monoamine Metabolism | 4.365109114 |
| Cloperastine hydrochloride | Monoamine Metabolism | 4.295466207 |
| Trifluoperazine dihydrochloride | Monoamine Metabolism | 4.242039959 |
| Pimethixene maleate | Monoamine Metabolism | 4.180505381 |
| Lanatoside C | Electrolyte Homeostasis | 4.159356048 |
| Oxyphenbutazone | Misc | 4.132349383 |
| Loracarbef | Antimicrobial | 4.132349383 |
| (S)-propranolol hydrochloride | Monoamine Metabolism | 4.043332095 |
| Maprotiline hydrochloride | Monoamine Metabolism | 3.96816181 |
| Albendazole | Antimicrobial | 3.965134358 |
| Imipramine hydrochloride | Monoamine Metabolism | 3.913427966 |
| GBR 12909 dihydrochloride | Monoamine Metabolism | 3.798310047 |
| **Securinine** | Monoamine Metabolism | 3.734163865 |
| Fluphenazine dihydrochloride | Monoamine Metabolism | 3.657461469 |
| Thioridazine hydrochloride | Monoamine Metabolism | 3.620900314 |
| **Penbutolol sulfate** | Monoamine Metabolism | 3.531482693 |
| Tetrandrine | Electrolyte Homeostasis | 3.468699447 |
| Dubinidine | Misc | 3.464719728 |
| Doxorubicin hydrochloride | DNA Damage | 3.463026667 |
| Palmatine chloride | Monoamine Metabolism | 3.408986588 |
| Netilmicin sulfate | Antimicrobial | 3.353448119 |
| Parthenolide | Misc | 3.310854505 |
| Acetopromazine maleate salt | Monoamine Metabolism | 3.306460676 |
| **Trimeprazine tartrate** | Monoamine Metabolism | 3.2754048 |
| Piperacetazine | Misc | 3.264430832 |
| Oxybutynin chloride | Anticholinergic | 3.257486713 |
| Paroxetine Hydrochloride | Monoamine Metabolism | 3.248418972 |
| Xylazine | Monoamine Metabolism | 3.203934763 |
| Econazole nitrate | Antimicrobial | 3.195952136 |
| Pimozide | Monoamine Metabolism | 3.165184847 |
| Spaglumic acid | Monoamine Metabolism | 3.153159223 |
| Perhexiline maleate | Misc | 3.103650269 |
| **Guanabenz acetate** | Monoamine Metabolism | 3.049616826 |
| Convolamine hydrochloride | Misc | 2.998882938 |
| Mecamylamine hydrochloride | Anticholinergic | 2.99737897 |
| (R)-Propranolol hydrochloride | Monoamine Metabolism | 2.975124648 |
| Diphenylpyraline hydrochloride | Anticholinergic | 2.926040689 |
| (-)-Quinpirole hydrochloride | Monoamine Metabolism | 2.841598717 |
| Corynanthine hydrochloride | Monoamine Metabolism | 2.793831114 |
| Quipazine dimaleate salt | Monoamine Metabolism | 2.793831114 |
| Monobenzone | Misc | 2.761458449 |
| Methapyrilene hydrochloride | Monoamine Metabolism | 2.745229117 |
| Hycanthone | Antimicrobial | 2.711258765 |
| Viomycin sulfate | Antimicrobial | 2.708072786 |
| Hydroquinine hydrobromide hydrate | Antimicrobial | 2.691305201 |
| Oxprenolol hydrochloride | Misc | 2.685818464 |
| Primaquine diphosphate | Antimicrobial | 2.674719669 |
| Fluspirilen | Monoamine Metabolism | 2.632581438 |
| Ethopropazine hydrochloride | Monoamine Metabolism | 2.627744942 |
| Dihydroergocristine mesylate | Monoamine Metabolism | 2.588779289 |
| Bromocryptine mesylate | Monoamine Metabolism | 2.587276027 |
| Midecamycin | Antimicrobial | 2.564722817 |
| 6-Furfurylaminopurine | Misc | 2.531379774 |
| Clorgyline hydrochloride | Monoamine Metabolism | 2.439495944 |
| Disulfiram | Misc | 2.418126954 |
| Haloperidol | Monoamine Metabolism | 2.406850349 |
| Fendiline hydrochloride | Electrolyte Homeostasis | 2.365235335 |
| **Sanguinarine** | Oxidative Stress | 2.349227947 |
| Hydroxyzine dihydrochloride | Monoamine Metabolism | 2.33919072 |
| Proadifen hydrochloride | Misc | 2.334673122 |
| Procarbazine hydrochloride | DNA Damage | 2.307494993 |
| Chlorpheniramine maleate | Monoamine Metabolism | 2.294084301 |
| Seneciphylline | DNA Damage | 2.281201552 |
| Fenspiride hydrochloride | Monoamine Metabolism | 2.236538424 |
| Perphenazine | Monoamine Metabolism | 2.215148067 |

Supplementary Table 1. The top 100 Prestwick Library drugs and small molecules that inhibit stress granule dissolution in U251 GBM cells induced by <1% hypoxia listed by Z-score and mechanism of action. Drugs listed in red promoted stress granule formation in normoxia and drug in green is a positive control.
