# Supplementary figures and images for: Raloxifene prevents stress granule dissolution, impairs translational control and promotes cell death during hypoxia in glioblastoma cells
Source: Cell Death Dis. 2020 Nov 17;11(11):989. doi: 10.1038/s41419-020-03159-5 (PMC7673037; doi:10.1038/s41419-020-03159-5)

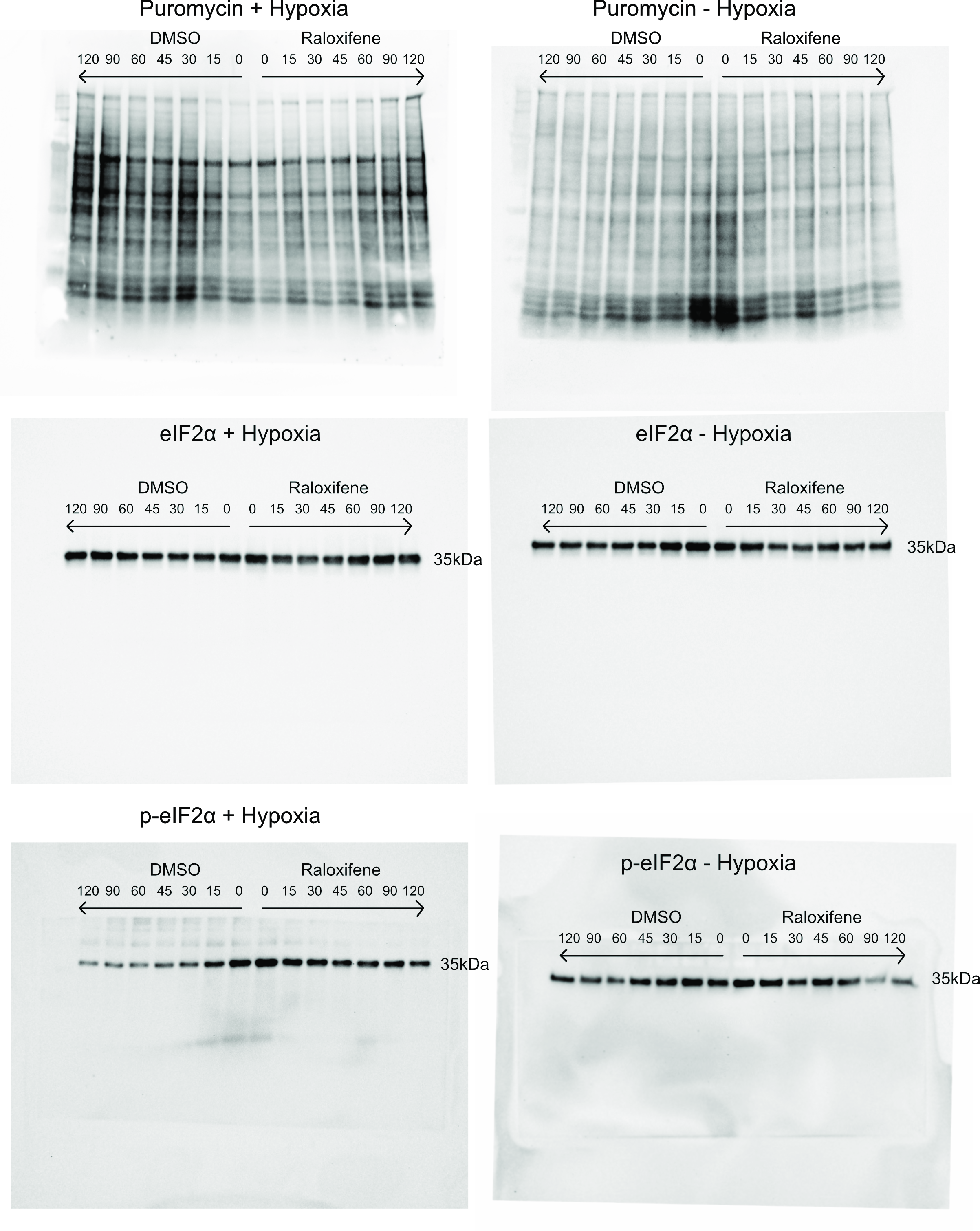

Supplement: Supplementary file 2 — Supplemental Figure 1 [file 41419_2020_3159_MOESM2_ESM.tif]

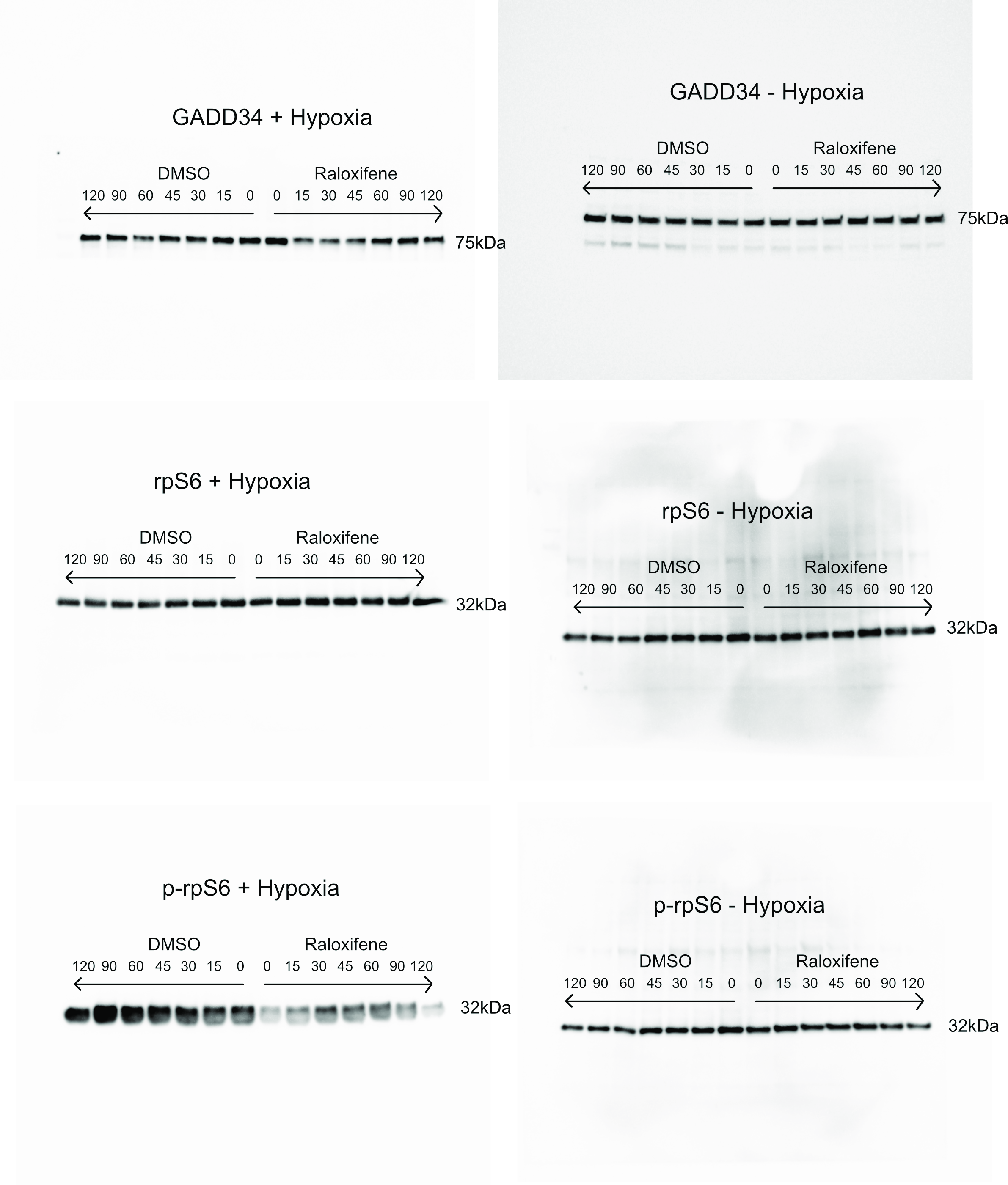

Supplement: Supplementary file 3 — Supplemental Figure 2 [file 41419_2020_3159_MOESM3_ESM.tif]

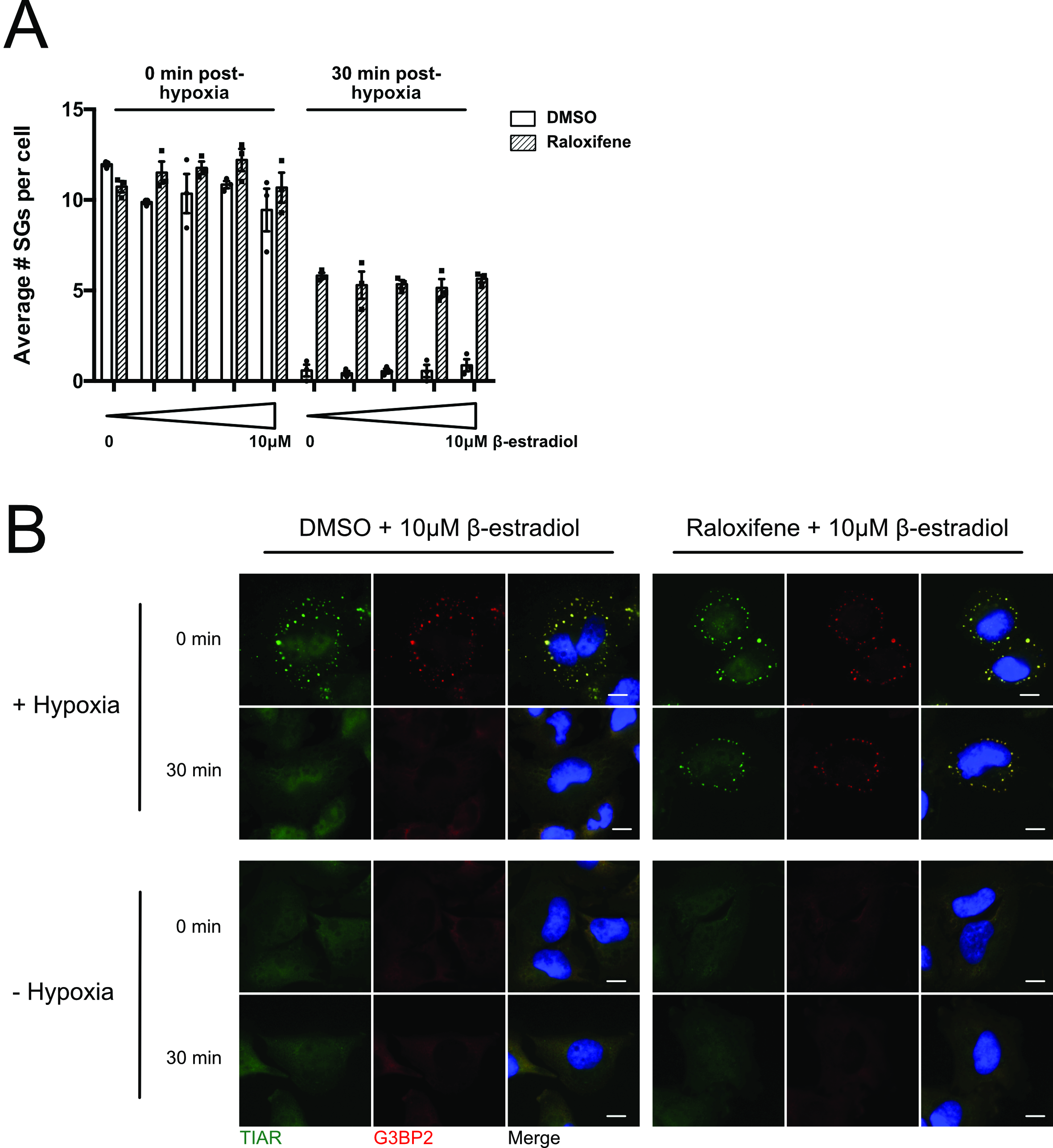

Supplement: Supplementary file 4 — Supplemental Figure 3 [file 41419_2020_3159_MOESM4_ESM.tif]

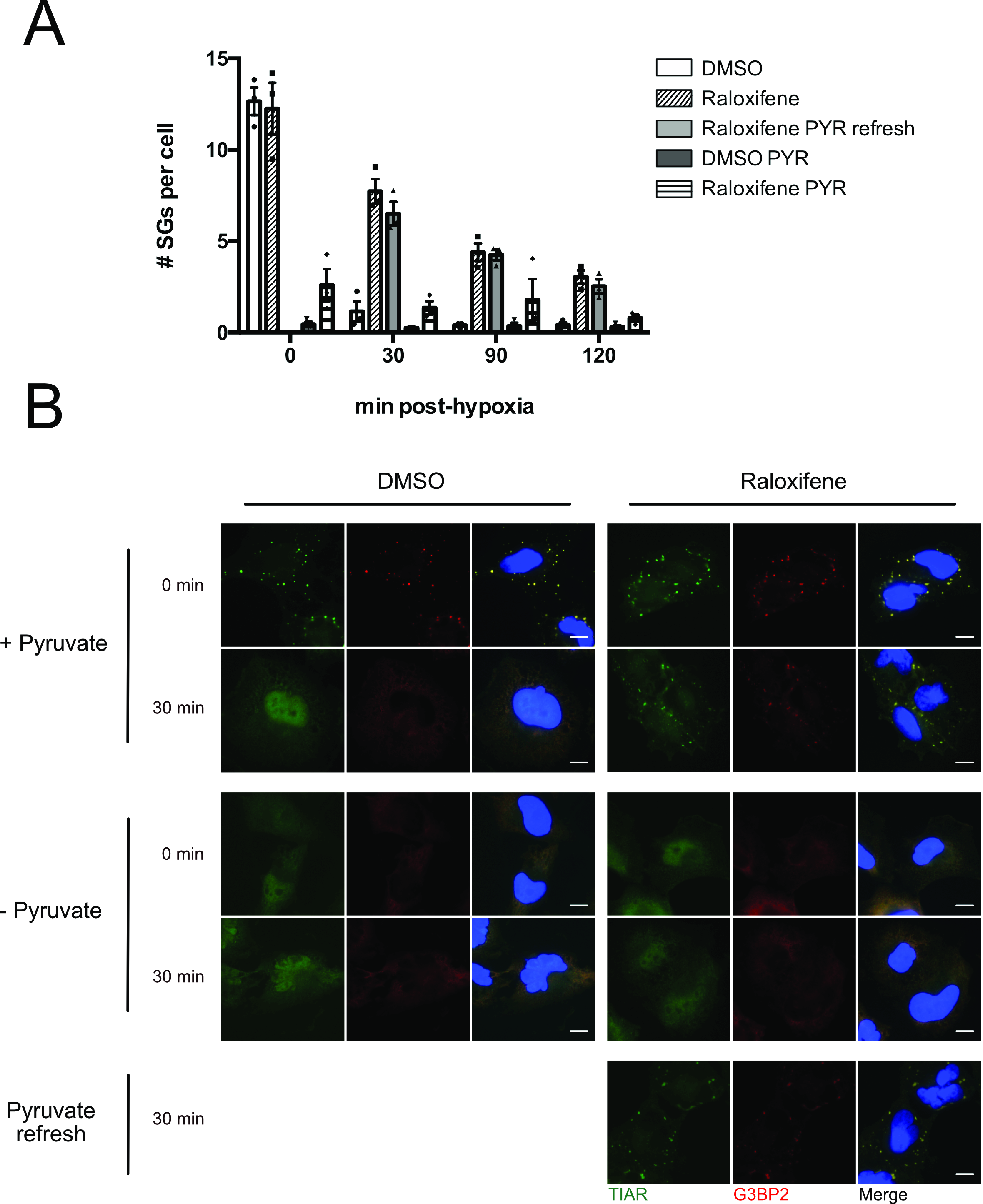

Supplement: Supplementary file 5 — Supplemental Figure 4 [file 41419_2020_3159_MOESM5_ESM.tif]
