# Supplementary material for: Raloxifene prevents stress granule dissolution, impairs translational control and promotes cell death during hypoxia in glioblastoma cells
Source: Cell Death Dis. 2020 Nov 17;11(11):989. doi: 10.1038/s41419-020-03159-5 (PMC7673037; doi:10.1038/s41419-020-03159-5)
Supplement: Supplementary file 7 — Supplemental Figure Legends [file 41419_2020_3159_MOESM7_ESM.docx]

**Supplemental Figure 1.** Whole western blot images from Figure 5. DMSO lanes were loaded 120→0min left to right while raloxifene lanes were loaded 0→120 left to right. Images in Figure 5 were cropped and rotated for easier visualization (**A**) Puromycin + hypoxia (**B**) Puromycin – hypoxia (**C**) eIF2α + hypoxia (**D**) eIF2α – hypoxia (**E**) p-eIF2α + hypoxia (**F**) p-eIF2α – hypoxia.

**Supplemental Figure 2.** Whole western blot images from Figure 5. DMSO lanes were loaded 120→0min left to right while raloxifene lanes were loaded 0→120 left to right. Images in Figure 5 were cropped and rotated for easier visualization (**A**) GADD34 + hypoxia (**B**) GADD34 – hypoxia (**C**) rpS6 + hypoxia (**D**) rpS6 – hypoxia (**E**) p-rpS6 + hypoxia (**F**) p-rpS6 – hypoxia.

**Supplemental Figure 3. The raloxifene-induced delay of SG dissolution is not mediated by ER signalling.** (**A**) U251 cells were treated with 40μM raloxifene or DMSO vehicle control along with increasing concentrations of β-estradiol (10nM, 100nM, 1μM, 10μM) for 1 h prior to 2 h of hypoxia (<1% O_2_). Cells were fixed either immediately (0 min) or 30 min post-hypoxia and stained for CellProfiler analysis as previously described. Data is presented as the mean of triplicates ± SEM. (**B**) Representative immunofluorescence images of (A) with 10μM β-estradiol ± hypoxia. Cells were stained with TIAR (green) and G3BP2 (red). DNA was counterstained with DAPI (blue). Scale bars = 10 microns.

**Supplemental Figure 4. The raloxifene-induced delay of SG dissolution is not due to prolonged oxidative stress.** (**A**) U251 cells were treated with 40μM raloxifene or DMSO vehicle control ± DMEM containing pyruvate (PYR) for 1 h prior to 2 h of hypoxia (<1% O_2_). Cells were fixed either immediately (0 min) or at various times post-hypoxia. A duplicate set of raloxifene treated cells were given fresh raloxifene/pyruvate media immediately post-hypoxia and were also fixed at the same time points. Cells were stained for CellProfiler analysis as previously described. Data is presented as the mean of triplicates ± SEM. (**B**) Representative immunofluorescence images of (A). Cells were stained with TIAR (green) and G3BP2 (red). DNA was counterstained with DAPI (blue). Scale bars = 10 microns.

**Supplemental Figure 5.** Whole western blot images from Figures 6 and 7. DMSO lanes were loaded 120→0min left to right while raloxifene or chloroquine lanes were loaded 0→120 left to right. Images in Figures 6 and 7 were cropped and rotated for easier visualization (**A**) LC3B + hypoxia raloxifene (**B**) LC3B - hypoxia raloxifene (**C**) p62 + hypoxia raloxifene (**D**) p62 - hypoxia raloxifene (**E**) LC3B + hypoxia chloroquine (**F**) LC3B - hypoxia chloroquine (**G**) p62 + hypoxia chloroquine (**H**) p62 - hypoxia chloroquine.
