# Supplementary material for: Raloxifene prevents stress granule dissolution, impairs translational control and promotes cell death during hypoxia in glioblastoma cells
Source: Cell Death Dis. 2020 Nov 17;11(11):989. doi: 10.1038/s41419-020-03159-5 (PMC7673037; doi:10.1038/s41419-020-03159-5)
Supplement: Supplementary file 8 — Supplemental Materials and Methods [file 41419_2020_3159_MOESM8_ESM.docx]

**Supplemental Materials and Methods**

**R-Code for Cell Profiler**

install.packages("tidyverse")

install.packages("pracma")

install.packages("plyr")

library("tidyverse")

library("pracma")

library("plyr")

library("tibble")

###open the folder where you'll find your files that you want to analyse

##bottom right corner, click file, find the folder, then 'In More', click 'set as working directory'

setwd(...)

#read the files and save them as dataframes

dfcytoplasm = read.csv("June 26_hyp_rep1_Cytoplasm.csv", header = T)

dfnuclei = read.csv("June 26_hyp_rep1_Nuclei.csv", header = T)

dfnames = read.csv("June 26_hyp_rep1_Image.csv", header = T)

dftiar = read.csv("June 26_hyp_rep1_tiar_gran.csv", header = T)

dfg3bp2 = read.csv("June 26_hyp_rep1_g3bp2_gran.csv", header = T)

dfcell = read.csv("June 26_hyp_rep1_Cells.csv", header = T)

###choose the columns that you'll need to analyze

###and set up the dataframes that you need for analysis

subsetdfcytoplasm = subset(dfcytoplasm, select= c("ImageNumber"

, "ObjectNumber"

, "Children_tiar_gran_Count"

, "Children_g3bp2_gran_Count"

, "Parent_Nuclei"))

subsetdfnuclei = subset(dfnuclei, select = c("ImageNumber",

"ObjectNumber",

"Children_Cytoplasm_Count"))

subsetdfnames = subset(dfnames, select = c("ImageNumber",

"Count_Cells",

"FileName_Dapi",

"Count_tiar_gran",

"Count_g3bp2_gran"))

subsetdftiargransize = subset(dftiar, select= c("ImageNumber"

, "ObjectNumber"

, "Parent_Nuclei"

, "AreaShape_Area"

, "AreaShape_MeanRadius"

, "Intensity_IntegratedIntensity_Cytoplasmictiar"))

subsetdfg3bp2gransize = subset(dfg3bp2, select= c("ImageNumber"

, "ObjectNumber"

, "Parent_Nuclei"

, "AreaShape_Area"

, "AreaShape_MeanRadius"

, "Intensity_IntegratedIntensity_Cytoplasmicg3bp2"))

subsetdfNloc = subset(dfnuclei, select = c("ImageNumber",

"ObjectNumber",

"Children_Cytoplasm_Count",

"Location_Center_X",

"Location_Center_Y"))

subsetdftiarloc = subset(dftiar, select = c("ImageNumber",

"ObjectNumber",

"Location_Center_X",

"Location_Center_Y"

, "Parent_Cytoplasm"))

subsetdfg3bp2loc = subset(dfg3bp2, select = c("ImageNumber",

"ObjectNumber",

"Location_Center_X",

"Location_Center_Y"

, "Parent_Cytoplasm"))

###rename some of the columns, so later on we can join the dataframes together without issue

subsetdfNloc = plyr::rename(subsetdfNloc, c("ObjectNumber" = "Parent_Cytoplasm"

, "Location_Center_X" = "Nuclei_Center_X"

, "Location_Center_Y" = "Nuclei_Center_Y"))

subsetdftiarloc = plyr::rename(subsetdftiarloc, c("ObjectNumber" = "SGnumber"

, "Location_Center_X" = "SG_Center_X"

, "Location_Center_Y" = "SG_Center_Y"))

subsetdfg3bp2loc = plyr::rename(subsetdfg3bp2loc, c("ObjectNumber" = "SGnumber"

, "Location_Center_X" = "g3bp2_Center_X"

, "Location_Center_Y" = "g3bp2_Center_Y"))

###count number of cells in each treatment

###first, make a column called file factor, with the bits of the image file names that tell

###you what each treatment is

###note: the number,number is the number of characters I want the file to read to distinguish files

subsetdfnames$filefactor = substr(subsetdfnames$FileName_Dapi, 1, 13)

###count the number of cells in each treatment

sumu251_HYP_D_00 = sum(subsetdfnames$Count_Cells[subsetdfnames$filefactor == "u251_HYP_D_00"])

sumu251_HYP_D_02 = sum(subsetdfnames$Count_Cells[subsetdfnames$filefactor == "u251_HYP_D_02"])

sumu251_HYP_D_15 = sum(subsetdfnames$Count_Cells[subsetdfnames$filefactor == "u251_HYP_D_15"])

sumu251_HYP_D_30 = sum(subsetdfnames$Count_Cells[subsetdfnames$filefactor == "u251_HYP_D_30"])

sumu251_HYP_D_45 = sum(subsetdfnames$Count_Cells[subsetdfnames$filefactor == "u251_HYP_D_45"])

sumu251_HYP_D_60 = sum(subsetdfnames$Count_Cells[subsetdfnames$filefactor == "u251_HYP_D_60"])

sumu251_HYP_D_90 = sum(subsetdfnames$Count_Cells[subsetdfnames$filefactor == "u251_HYP_D_90"])

sumu251_HYP_R_00 = sum(subsetdfnames$Count_Cells[subsetdfnames$filefactor == "u251_HYP_R_00"])

sumu251_HYP_R_02 = sum(subsetdfnames$Count_Cells[subsetdfnames$filefactor == "u251_HYP_R_02"])

sumu251_HYP_R_15 = sum(subsetdfnames$Count_Cells[subsetdfnames$filefactor == "u251_HYP_R_15"])

sumu251_HYP_R_30 = sum(subsetdfnames$Count_Cells[subsetdfnames$filefactor == "u251_HYP_R_30"])

sumu251_HYP_R_45 = sum(subsetdfnames$Count_Cells[subsetdfnames$filefactor == "u251_HYP_R_45"])

sumu251_HYP_R_60 = sum(subsetdfnames$Count_Cells[subsetdfnames$filefactor == "u251_HYP_R_60"])

sumu251_HYP_R_90 = sum(subsetdfnames$Count_Cells[subsetdfnames$filefactor == "u251_HYP_R_90"])

#add treatment labels to the other files

subsetdfcytoplasm$Treatment = rep(c("u251_HYP_D_00",

"u251_HYP_D_02",

"u251_HYP_D_15",

"u251_HYP_D_30",

"u251_HYP_D_45",

"u251_HYP_D_60",

"u251_HYP_D_90",

"u251_HYP_R_00",

"u251_HYP_R_02",

"u251_HYP_R_15",

"u251_HYP_R_30",

"u251_HYP_R_45",

"u251_HYP_R_60",

"u251_HYP_R_90")

, times = c(sumu251_HYP_D_00,

sumu251_HYP_D_02,

sumu251_HYP_D_15,

sumu251_HYP_D_30,

sumu251_HYP_D_45,

sumu251_HYP_D_60,

sumu251_HYP_D_90,

sumu251_HYP_R_00,

sumu251_HYP_R_02,

sumu251_HYP_R_15,

sumu251_HYP_R_30,

sumu251_HYP_R_45,

sumu251_HYP_R_60,

sumu251_HYP_R_90))

subsetdfnuclei$Treatment = rep(c("u251_HYP_D_00",

"u251_HYP_D_02",

"u251_HYP_D_15",

"u251_HYP_D_30",

"u251_HYP_D_45",

"u251_HYP_D_60",

"u251_HYP_D_90",

"u251_HYP_R_00",

"u251_HYP_R_02",

"u251_HYP_R_15",

"u251_HYP_R_30",

"u251_HYP_R_45",

"u251_HYP_R_60",

"u251_HYP_R_90")

, times = c(sumu251_HYP_D_00,

sumu251_HYP_D_02,

sumu251_HYP_D_15,

sumu251_HYP_D_30,

sumu251_HYP_D_45,

sumu251_HYP_D_60,

sumu251_HYP_D_90,

sumu251_HYP_R_00,

sumu251_HYP_R_02,

sumu251_HYP_R_15,

sumu251_HYP_R_30,

sumu251_HYP_R_45,

sumu251_HYP_R_60,

sumu251_HYP_R_90))

subsetdfNloc$Treatment = rep(c("u251_HYP_D_00",

"u251_HYP_D_02",

"u251_HYP_D_15",

"u251_HYP_D_30",

"u251_HYP_D_45",

"u251_HYP_D_60",

"u251_HYP_D_90",

"u251_HYP_R_00",

"u251_HYP_R_02",

"u251_HYP_R_15",

"u251_HYP_R_30",

"u251_HYP_R_45",

"u251_HYP_R_60",

"u251_HYP_R_90")

, times = c(sumu251_HYP_D_00,

sumu251_HYP_D_02,

sumu251_HYP_D_15,

sumu251_HYP_D_30,

sumu251_HYP_D_45,

sumu251_HYP_D_60,

sumu251_HYP_D_90,

sumu251_HYP_R_00,

sumu251_HYP_R_02,

sumu251_HYP_R_15,

sumu251_HYP_R_30,

sumu251_HYP_R_45,

sumu251_HYP_R_60,

sumu251_HYP_R_90))

#save cell counts

cellcounts = c(sumu251_HYP_D_00,

sumu251_HYP_D_02,

sumu251_HYP_D_15,

sumu251_HYP_D_30,

sumu251_HYP_D_45,

sumu251_HYP_D_60,

sumu251_HYP_D_90,

sumu251_HYP_R_00,

sumu251_HYP_R_02,

sumu251_HYP_R_15,

sumu251_HYP_R_30,

sumu251_HYP_R_45,

sumu251_HYP_R_60,

sumu251_HYP_R_90)

cellcounts = data.frame(cellcounts)

cellcounts$Treatment = c("u251_HYP_D_00",

"u251_HYP_D_02",

"u251_HYP_D_15",

"u251_HYP_D_30",

"u251_HYP_D_45",

"u251_HYP_D_60",

"u251_HYP_D_90",

"u251_HYP_R_00",

"u251_HYP_R_02",

"u251_HYP_R_15",

"u251_HYP_R_30",

"u251_HYP_R_45",

"u251_HYP_R_60",

"u251_HYP_R_90")

write.csv(cellcounts, file = "June 26_hyp_rep1_cellcounts.csv")

###same thing for the number of SGs

sumu251_HYP_D_00tiargran = sum(subsetdfnames$Count_tiar_gran[subsetdfnames$filefactor == "u251_HYP_D_00"])

sumu251_HYP_D_02tiargran = sum(subsetdfnames$Count_tiar_gran[subsetdfnames$filefactor == "u251_HYP_D_02"])

sumu251_HYP_D_15tiargran = sum(subsetdfnames$Count_tiar_gran[subsetdfnames$filefactor == "u251_HYP_D_15"])

sumu251_HYP_D_30tiargran = sum(subsetdfnames$Count_tiar_gran[subsetdfnames$filefactor == "u251_HYP_D_30"])

sumu251_HYP_D_45tiargran = sum(subsetdfnames$Count_tiar_gran[subsetdfnames$filefactor == "u251_HYP_D_45"])

sumu251_HYP_D_60tiargran = sum(subsetdfnames$Count_tiar_gran[subsetdfnames$filefactor == "u251_HYP_D_60"])

sumu251_HYP_D_90tiargran = sum(subsetdfnames$Count_tiar_gran[subsetdfnames$filefactor == "u251_HYP_D_90"])

sumu251_HYP_R_00tiargran = sum(subsetdfnames$Count_tiar_gran[subsetdfnames$filefactor == "u251_HYP_R_00"])

sumu251_HYP_R_02tiargran = sum(subsetdfnames$Count_tiar_gran[subsetdfnames$filefactor == "u251_HYP_R_02"])

sumu251_HYP_R_15tiargran = sum(subsetdfnames$Count_tiar_gran[subsetdfnames$filefactor == "u251_HYP_R_15"])

sumu251_HYP_R_30tiargran = sum(subsetdfnames$Count_tiar_gran[subsetdfnames$filefactor == "u251_HYP_R_30"])

sumu251_HYP_R_45tiargran = sum(subsetdfnames$Count_tiar_gran[subsetdfnames$filefactor == "u251_HYP_R_45"])

sumu251_HYP_R_60tiargran = sum(subsetdfnames$Count_tiar_gran[subsetdfnames$filefactor == "u251_HYP_R_60"])

sumu251_HYP_R_90tiargran = sum(subsetdfnames$Count_tiar_gran[subsetdfnames$filefactor == "u251_HYP_R_90"])

subsetdftiargransize$Treatment = rep(c("u251_HYP_D_00",

"u251_HYP_D_02",

"u251_HYP_D_15",

"u251_HYP_D_30",

"u251_HYP_D_45",

"u251_HYP_D_60",

"u251_HYP_D_90",

"u251_HYP_R_00",

"u251_HYP_R_02",

"u251_HYP_R_15",

"u251_HYP_R_30",

"u251_HYP_R_45",

"u251_HYP_R_60",

"u251_HYP_R_90")

, times = c(sumu251_HYP_D_00tiargran,

sumu251_HYP_D_02tiargran,

sumu251_HYP_D_15tiargran,

sumu251_HYP_D_30tiargran,

sumu251_HYP_D_45tiargran,

sumu251_HYP_D_60tiargran,

sumu251_HYP_D_90tiargran,

sumu251_HYP_R_00tiargran,

sumu251_HYP_R_02tiargran,

sumu251_HYP_R_15tiargran,

sumu251_HYP_R_30tiargran,

sumu251_HYP_R_45tiargran,

sumu251_HYP_R_60tiargran,

sumu251_HYP_R_90tiargran))

sumu251_HYP_D_00g3bp2gran = sum(subsetdfnames$Count_g3bp2_gran[subsetdfnames$filefactor == "u251_HYP_D_00"])

sumu251_HYP_D_02g3bp2gran = sum(subsetdfnames$Count_g3bp2_gran[subsetdfnames$filefactor == "u251_HYP_D_02"])

sumu251_HYP_D_15g3bp2gran = sum(subsetdfnames$Count_g3bp2_gran[subsetdfnames$filefactor == "u251_HYP_D_15"])

sumu251_HYP_D_30g3bp2gran = sum(subsetdfnames$Count_g3bp2_gran[subsetdfnames$filefactor == "u251_HYP_D_30"])

sumu251_HYP_D_45g3bp2gran = sum(subsetdfnames$Count_g3bp2_gran[subsetdfnames$filefactor == "u251_HYP_D_45"])

sumu251_HYP_D_60g3bp2gran = sum(subsetdfnames$Count_g3bp2_gran[subsetdfnames$filefactor == "u251_HYP_D_60"])

sumu251_HYP_D_90g3bp2gran = sum(subsetdfnames$Count_g3bp2_gran[subsetdfnames$filefactor == "u251_HYP_D_90"])

sumu251_HYP_R_00g3bp2gran = sum(subsetdfnames$Count_g3bp2_gran[subsetdfnames$filefactor == "u251_HYP_R_00"])

sumu251_HYP_R_02g3bp2gran = sum(subsetdfnames$Count_g3bp2_gran[subsetdfnames$filefactor == "u251_HYP_R_02"])

sumu251_HYP_R_15g3bp2gran = sum(subsetdfnames$Count_g3bp2_gran[subsetdfnames$filefactor == "u251_HYP_R_15"])

sumu251_HYP_R_30g3bp2gran = sum(subsetdfnames$Count_g3bp2_gran[subsetdfnames$filefactor == "u251_HYP_R_30"])

sumu251_HYP_R_45g3bp2gran = sum(subsetdfnames$Count_g3bp2_gran[subsetdfnames$filefactor == "u251_HYP_R_45"])

sumu251_HYP_R_60g3bp2gran = sum(subsetdfnames$Count_g3bp2_gran[subsetdfnames$filefactor == "u251_HYP_R_60"])

sumu251_HYP_R_90g3bp2gran = sum(subsetdfnames$Count_g3bp2_gran[subsetdfnames$filefactor == "u251_HYP_R_90"])

subsetdfg3bp2gransize$Treatment = rep(c("u251_HYP_D_00",

"u251_HYP_D_02",

"u251_HYP_D_15",

"u251_HYP_D_30",

"u251_HYP_D_45",

"u251_HYP_D_60",

"u251_HYP_D_90",

"u251_HYP_R_00",

"u251_HYP_R_02",

"u251_HYP_R_15",

"u251_HYP_R_30",

"u251_HYP_R_45",

"u251_HYP_R_60",

"u251_HYP_R_90")

, times = c(sumu251_HYP_D_00g3bp2gran,

sumu251_HYP_D_02g3bp2gran,

sumu251_HYP_D_15g3bp2gran,

sumu251_HYP_D_30g3bp2gran,

sumu251_HYP_D_45g3bp2gran,

sumu251_HYP_D_60g3bp2gran,

sumu251_HYP_D_90g3bp2gran,

sumu251_HYP_R_00g3bp2gran,

sumu251_HYP_R_02g3bp2gran,

sumu251_HYP_R_15g3bp2gran,

sumu251_HYP_R_30g3bp2gran,

sumu251_HYP_R_45g3bp2gran,

sumu251_HYP_R_60g3bp2gran,

sumu251_HYP_R_90g3bp2gran))

###calculate the percent cells - note: you need to detach the plyr package, the reattach

detach(package:plyr)

percentcellswithtiargran = subsetdfcytoplasm %>%

group_by(Treatment) %>%

summarise (mean(Children_tiar_gran_Count > 0))

percentcellswithg3bp2gran = subsetdfcytoplasm %>%

group_by(Treatment) %>%

summarise (mean(Children_g3bp2_gran_Count > 0))

library(plyr)

###save the file

percentcellswithtiargran$Percent = percentcellswithtiargran$"mean(Children_tiar_gran_Count > 0)"*100

write.csv(percentcellswithtiargran, file = "June 26_hyp_rep1_percentcellswithtiargran.csv")

percentcellswithg3bp2gran$Percent = percentcellswithg3bp2gran$"mean(Children_g3bp2_gran_Count > 0)"*100

write.csv(percentcellswithg3bp2gran, file = "June 26_hyp_rep1_percentcellswithg3bp2gran.csv")

##calculate the number of SGs per cell

dfmeantiarcount <-

aggregate(

formula = Children_tiar_gran_Count ~ Treatment

, data = subsetdfcytoplasm

, FUN = mean

)

write.csv(dfmeantiarcount, file = "June 26_hyp_rep1_dfmeantiarcount.csv")

dfmeang3bp2count <-

aggregate(

formula = Children_g3bp2_gran_Count ~ Treatment

, data = subsetdfcytoplasm

, FUN = mean

)

write.csv(dfmeang3bp2count, file = "June 26_hyp_rep1_dfmeang3bp2count.csv")

###calculate the number of SGs per cell without those that contain 0

subsetdfcytoplasm %>%

dplyr::filter(Children_tiar_gran_Count > 0) %>%

group_by(Treatment) %>%

dplyr::summarise(Children_tiar_gran_Count = mean(Children_tiar_gran_Count)) -> dfmeantiargrancountwithout0

write.csv(dfmeantiargrancountwithout0, file = "June 26_hyp_rep1_dfmeantiargrancountwithout0.csv")

subsetdfcytoplasm %>%

dplyr::filter(Children_g3bp2_gran_Count > 0) %>%

group_by(Treatment) %>%

dplyr::summarise(Children_g3bp2_gran_Count = mean(Children_g3bp2_gran_Count)) -> dfmeang3bp2grancountwithout0

write.csv(dfmeang3bp2grancountwithout0, file = "June 26_hyp_rep1_dfmeang3bp2grancountwithout0.csv")

###averagesize of SGs, looking at pixel size

subsetdftiargransize$Size = subsetdftiargransize$AreaShape_MeanRadius*420

dfmeantiargransize =

aggregate(

formula = Size ~ Treatment

, data = subsetdftiargransize

, FUN = mean

)

write.csv(dfmeantiargransize, file = "June 26_hyp_rep1_dfmeantiargransize.csv")

subsetdfg3bp2gransize$Size = subsetdfg3bp2gransize$AreaShape_MeanRadius*420

dfmeang3bp2gransize =

aggregate(

formula = Size ~ Treatment

, data = subsetdfg3bp2gransize

, FUN = mean

)

write.csv(dfmeang3bp2gransize, file = "June 26_hyp_rep1_dfmeang3bp2gransize.csv")

#sizeviaSGintensity

dfmeantiargranintensity =

aggregate(

formula = Intensity_IntegratedIntensity_Cytoplasmictiar ~ Treatment

, data = subsetdftiargransize

, FUN = mean

)

write.csv(dfmeantiargranintensity, file = "June 26_hyp_rep1_dfmeantiargranintensity.csv")

dfmeang3bp2granintensity =

aggregate(

formula = Intensity_IntegratedIntensity_Cytoplasmicg3bp2 ~ Treatment

, data = subsetdfg3bp2gransize

, FUN = mean

)

write.csv(dfmeang3bp2granintensity, file = "June 26_hyp_rep1_dfmeang3bp2granintensity.csv")

#correlation between g3bp2 and tiar puncta

SGcorrelation = subset(dfg3bp2, select = c("ImageNumber",

"ObjectNumber",

"Correlation_Correlation_Cytoplasmicg3bp2_Cytoplasmictiar"))

#adding treatment variables

SGcorrelation = join(SGcorrelation, subsetdfg3bp2gransize)

##look at percentage of granules correlated per treatment

#find the number of SGs that have a higher correlation value than 0.5

SGcorrelationthresh = SGcorrelation[SGcorrelation$Correlation_Correlation_Cytoplasmicg3bp2_Cytoplasmictiar>0.50000000,]

#get rid of NA

SGcorrelationthresh = na.omit(SGcorrelationthresh)

#count the number of SGs after the threshold

SGcorrthreshcount = as.data.frame(table(SGcorrelationthresh$Treatment))

#count the number of SGs before the threshold

SGcorrcount = as.data.frame(table(SGcorrelation$Treatment))

#rename so we can put the two together and look at percentage

SGcorrthreshcount = plyr::rename(SGcorrthreshcount, c("Freq" = "ThreshFreq"))

#put the two together and then calculate the percentage

SGcorrcount = join(SGcorrcount, SGcorrthreshcount)

SGcorrcount$Percent = SGcorrcount$ThreshFreq/SGcorrcount$Freq

#put the percent to multiply granules by into the correct dataframe

subsetdfcytoplasm$percentcorr = rep(c(SGcorrcount$Percent)

, times = c(sumu251_HYP_D_00,

sumu251_HYP_D_02,

sumu251_HYP_D_15,

sumu251_HYP_D_30,

sumu251_HYP_D_45,

sumu251_HYP_D_60,

sumu251_HYP_D_90,

sumu251_HYP_R_00,

sumu251_HYP_R_02,

sumu251_HYP_R_15,

sumu251_HYP_R_30,

sumu251_HYP_R_45,

sumu251_HYP_R_60,

sumu251_HYP_R_90))

#multiply granule counts by percentage

subsetdfcytoplasm$corrSGcounts = subsetdfcytoplasm$Children_g3bp2_gran_Count*subsetdfcytoplasm$percentcorr

#redo measurements

###SG count per cell

#join data sets

dfmeanSGcountcorr <-

aggregate(

formula = corrSGcounts ~ Treatment

, data = subsetdfcytoplasm

, FUN = mean

)

write.csv(dfmeanSGcountcorr, file = "June 26_hyp_rep1_dfmeanSGcountcorr.csv")

subsetdfcytoplasm %>%

dplyr::filter(corrSGcounts > 0) %>%

group_by(Treatment) %>%

dplyr::summarise(x = mean(corrSGcounts)) -> dfmeanSGcountcorrwithout0

write.csv(dfmeanSGcountcorrwithout0, file = "June 26_hyp_rep1_dfmeanSGcountcorrwithout0.csv")

##number of cells th normal SGs

detach(package:plyr)

percentcellswithSGscorr = subsetdfcytoplasm %>%

group_by(Treatment) %>%

summarise (mean(corrSGcounts > 0))

library(plyr)

write.csv(percentcellswithSGscorr, file = "June 26_hyp_rep1_percentcellswithSGscorr.csv")

###average size of SGs

subsetdfSGsize$Size = subsetdfSGsize$AreaShape_MeanRadius*420

dfmeanSGsizecorr =

aggregate(

formula = Size ~ Treatment

, data = SGcorrelationthresh

, FUN = mean

)

write.csv(dfmeanSGsizecorr, file = "June 26_hyp_rep1_dfmeanSGsizecorr.csv")

##SG intensity

dfmeanSGintensitycorr =

aggregate(

formula = Intensity_IntegratedIntensity_Cytoplasmicg3bp2 ~ Treatment

, data = SGcorrelationthresh

, FUN = mean

)

write.csv(dfmeanSGintensitycorr, file = "June 26_hyp_rep1_dfmeanSGintensitycorr.csv")

##look at percentage of granules correlated per treatment

#find the number of SGs that have a higher correlation value than 0.5

SGintensitythresh = SGcorrelationthresh[SGcorrelationthresh$Intensity_IntegratedIntensity_Cytoplasmicg3bp2>0.5,]

#get rid of NA

SGintensitythresh = na.omit(SGintensitythresh)

#count the number of SGs after the threshold

SGintthreshcount = as.data.frame(table(SGintensitythresh$Treatment))

#rename so we can put the two together and look at percentage

SGintthreshcount = plyr::rename(SGintthreshcount, c("Freq" = "IntThreshFreq"))

#put the two together and then calculate the percentage

SGintcount = join(SGcorrcount, SGintthreshcount)

SGintcount$PercentInt = SGintcount$IntThreshFreq/SGintcount$ThreshFreq

#put the percent to multiply granules by into the correct dataframe

subsetdfcytoplasm$percentint = rep(c(SGintcount$PercentInt)

, times = c(sumu251_HYP_D_00,

sumu251_HYP_D_02,

sumu251_HYP_D_15,

sumu251_HYP_D_30,

sumu251_HYP_D_45,

sumu251_HYP_D_60,

sumu251_HYP_D_90,

sumu251_HYP_R_00,

sumu251_HYP_R_02,

sumu251_HYP_R_15,

sumu251_HYP_R_30,

sumu251_HYP_R_45,

sumu251_HYP_R_60,

sumu251_HYP_R_90))

#multiply granule counts by percentage intensity threshold

subsetdfcytoplasm$corr_int_SGcounts = subsetdfcytoplasm$corrSGcounts*subsetdfcytoplasm$percentint

#redo measurements

###SG count per cell

#join data sets

dfmeanSGcountint <-

aggregate(

formula = corr_int_SGcounts ~ Treatment

, data = subsetdfcytoplasm

, FUN = mean

)

write.csv(dfmeanSGcountint, file = "June 26_hyp_rep1_dfmeanSGcountint.csv")

subsetdfcytoplasm %>%

dplyr::filter(corr_int_SGcounts > 0) %>%

group_by(Treatment) %>%

dplyr::summarise(x = mean(corr_int_SGcounts)) -> dfmeanSGcountintwithout0

write.csv(dfmeanSGcountintwithout0, file = "June 26_hyp_rep1_dfmeanSGcountintwithout0.csv")

##number of cells with normal SGs

detach(package:plyr)

percentcellswithSGsint = subsetdfcytoplasm %>%

group_by(Treatment) %>%

summarise (mean(corr_int_SGcounts > 0))

library(plyr)

write.csv(percentcellswithSGsint, file = "June 26_hyp_rep1_percentcellswithSGsint.csv")

##SG intensity

dfmeanSGintensityint =

aggregate(

formula = Intensity_IntegratedIntensity_Cytoplasmicg3bp2 ~ Treatment

, data = SGintensitythresh

, FUN = mean

)

write.csv(dfmeanSGintensityint, file = "June 26_hyp_rep1_dfmeanSGintensityint.csv")
